# Proximity labeling reveals new functional relationships between meiotic recombination proteins in *S*. *cerevisiae*

**DOI:** 10.1371/journal.pgen.1011432

**Published:** 2024-10-15

**Authors:** Karen Voelkel-Meiman, Jennifer C. Liddle, Jeremy L. Balsbaugh, Amy J. MacQueen

**Affiliations:** 1 Department of Molecular Biology and Biochemistry, Wesleyan University, Middletown, Connecticut, United States of America; 2 Proteomics and Metabolomics Facility, Center for Open Research Resources and Equipment, University of Connecticut, Storrs, Connecticut, United States of America; National Cancer Institute, UNITED STATES OF AMERICA

## Abstract

Several protein ensembles facilitate crossover recombination and the associated assembly of synaptonemal complex (SC) during meiosis. In yeast, meiosis-specific factors including the DNA helicase Mer3, the “ZZS” complex consisting of Zip4, Zip2, and Spo16, the RING-domain protein Zip3, and the MutSγ heterodimer collaborate with crossover-promoting activity of the SC component, Zip1, to generate crossover-designated recombination intermediates. These ensembles also promote SC formation ‐ the organized assembly of Zip1 with other structural proteins between aligned chromosome axes. We used proximity labeling to investigate spatial relationships between meiotic recombination and SC proteins in *S*. *cerevisiae*. We find that recombination initiation and SC factors are dispensable for proximity labeling of Zip3 by ZZS components, but proteins associated with early steps in recombination are required for Zip3 proximity labeling by MutSγ, suggesting that MutSγ joins Zip3 only after a recombination intermediate has been generated. We also find that *zip1* separation-of-function mutants that are crossover deficient but still assemble SC fail to generate protein ensembles where Zip3 can engage ZZS and/or MutSγ. The SC structural protein Ecm11 is proximity labeled by ZZS proteins in a Zip4-dependent and Zip1-independent manner, but labeling of Ecm11 by Zip3 and MutSγ requires, at least in part, Zip1. Finally, mass spectrometry analysis of biotinylated proteins in eleven proximity labeling strains uncovered shared proximity targets of SC and crossover-associated proteins, some of which have not previously been implicated in meiotic recombination or SC formation, highlighting the potential of proximity labeling as a discovery tool.

## Author summary

Sexually reproducing organisms carry two homologous sets of chromosomes, one from each parent. A specialized cell division cycle, meiosis, generates reproductive cells with one complete chromosome set. The segregation of homologous chromosomes to opposite poles of the meiosis I spindle (the so-called reductional division) critically depends upon transient linkages formed in part through crossover recombination events, where homologous DNA duplexes undergo reciprocal exchange. Numerous proteins co-localize at chromosomal sites and promote the formation of crossovers as well as the assembly of a conserved protein-rich structure (the synaptonemal complex, SC) along the lengths of pairwise-aligned homologous chromosome axes. We employed proximity labeling to probe spatial and functional relationships between meiotic recombination and SC proteins in budding yeast. Proximity labeling involves the *in vivo* expression of a gene encoding a bait protein fused to a small biotinylase enzyme, and then the evaluation of nearby “target” proteins biotinylated by the fusion. The data we obtained for several meiotic bait proteins highlights how proximity labeling can be used as a phenotyping tool to determine underlying functional requirements for the formation of specific protein ensembles, and as a discovery tool to identify new potential components of recombination and SC assembly pathways.

## Introduction

Numerous proteins interact with one another and with DNA during meiosis to ensure that gametes contain the proper number of chromosomes. Homologous chromosomes (homologs) must segregate from one another at meiosis I, and this relies on prior establishment of crossover recombination-based linkages [1]. Interhomolog crossovers form during meiosis through coordinately acting groups of proteins that promote repair of programmed DNA double strand breaks (DSBs) via homologous recombination [2,3]. Crosstalk between DSB repair machinery and the chromosome axis somehow ensures that meiotic crossover events preferentially involve non-sister chromatids (i.e. a chromatid belonging to each homolog) and facilitates crossover patterning such that every homolog pair receives at least one crossover-mediated attachment.

**Fig 1:**
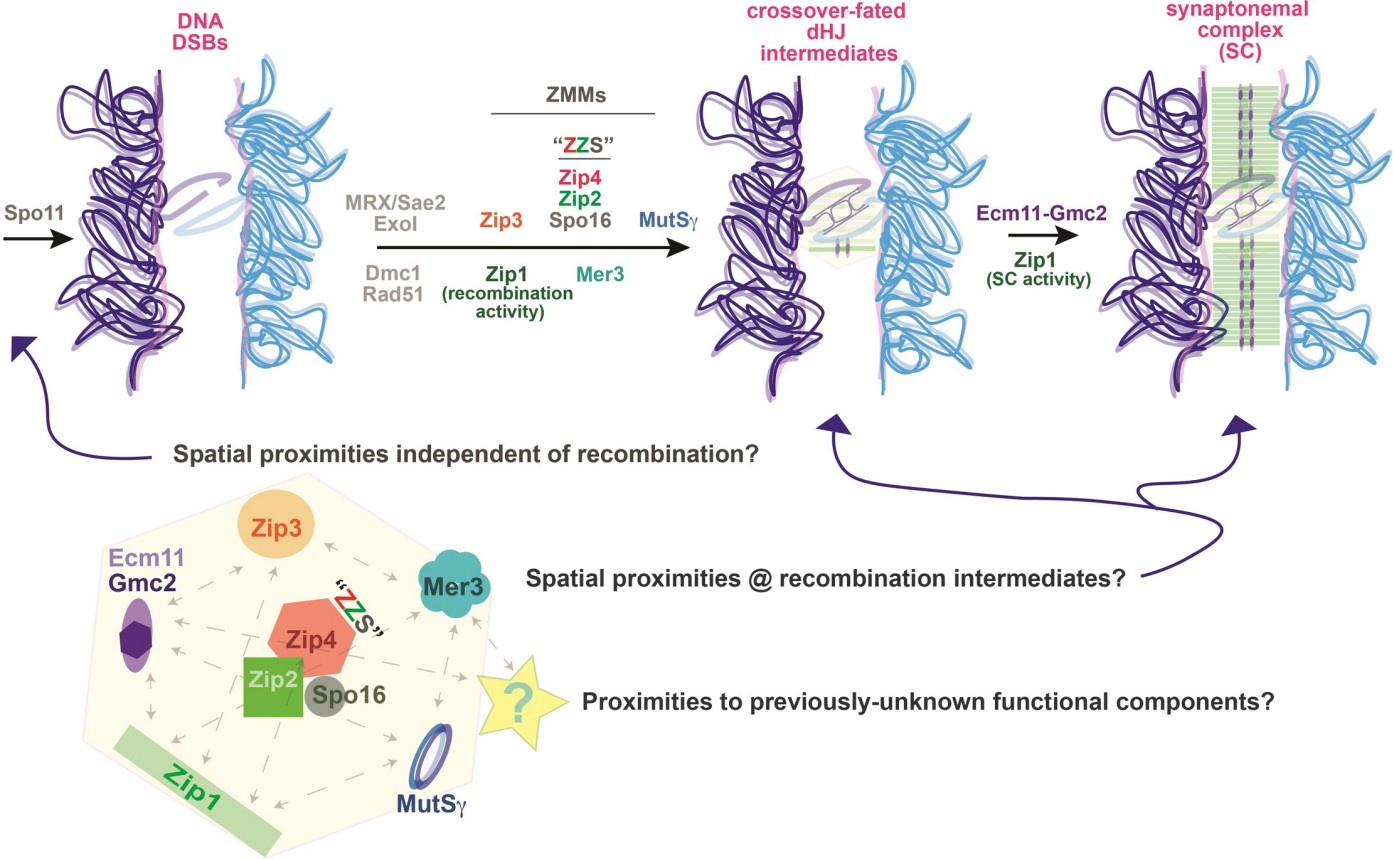
Many proteins facilitate the formation of crossover-designated recombination intermediates and synaptonemal complex (SC) assembly between homologs during meiosis. The cartoon illustrates key steps and proteins involved in meiotic recombination and SC assembly (synapsis) in *S*. *cerevisiae*. The chromatin of each partner chromosome in a homolog pair is indicated in dark and light blue; chromatin loops belonging to each sister chromatid are organized as an array anchored to a protein-rich axial core (indicated in light pink). A Spo11-mediated DNA double strand break (DSB) is processed via several enzymatic steps facilitated by proteins including MRX (Mre11, Rad50, Xrs2), Sae2, and Exo1. RecA proteins Dmc1 and Rad51 assemble a nucleoprotein filament that incorporates 3’ single-stranded DNA at the processed ends of a DSB, which is capable of strand invasion with a homologous duplex. With the help of ZMM proteins, a strand invasion intermediate can develop into a mature DNA joint molecule structure that contains a double Holliday junction. Cytological and ChIP studies suggest that Zip1 and Zip3 act early to stabilize the interaction of ZZS and MutSγ complexes at recombination intermediates to ensure dHJ formation, although Zip1 is not absolutely required for dHJ formation like the other ZMM proteins (Zip1’s crossover activity is required, however, for crossover-biased resolution of ZMM joint molecules). The illustration shows that SC assembly is triggered downstream of early steps in recombination, occurs at recombination intermediates, and requires the activity of most ZMM proteins. Also of importance is the positioning of dHJ recombination intermediates between homologous chromosome axes where SC central region components self-assemble. For clarity, only a shaded yellow hexagon represents ZMM protein ensembles at recombination intermediates. A larger yellow hexagon, illustrated below, contains individual ZMM and SC proteins, with dashed line-arrows reflecting uncertainty about the spatial arrangements of these factors prior to and during the dynamic processes of recombination and synapsis. Dashed arrows are not meant to indicate the existence of evidence for an interaction, but instead represent potential interactions that could occur during a meiotic process.

Several meiotic crossover-promoting factors have been identified and characterized to some degree (Fig 1). In *S*. *cerevisiae*, DNA DSBs are induced during meiosis by Spo11 and processed through multiple steps [4], ultimately leading to the formation of a Dmc1/Rad51-coated nucleoprotein filament competent for strand invasion with a homologous DNA duplex. Strand invasion intermediates develop into crossover-fated joint molecules (typically double Holliday junction structures) with the help of a mostly conserved group of proteins referred to as “ZMMs” [5]. ZMMs include the Mer3 DNA helicase [6–9], the ZZS complex consisting of Zip4 [10,11], Zip2 [12–15], and Spo16 [16,17], the RING-domain protein with E3 SUMO ligase activity, Zip3 [18–23], and the MutSγ (Msh4-Msh5) heterodimer [24–27]. Holliday junction-containing recombination intermediates and crossovers are dramatically reduced when any of these meiosis-specific factors is absent [16, 28]. Finally, MutLγ (Mlh1-Mlh3 heterodimer) and ExoI together resolve ZMM-associated joint molecules [29], processing the two junctions of an intermediate in a biased manner to ensure a crossover outcome [30].

Meiotic recombination is functionally linked to synapsis, where a supramolecular multi-protein structure called the synaptonemal complex (SC) assembles along the lengths of aligned homolog axes [31; Fig 1]. The central region of SC typically contains two structurally discernable features: Transverse filament proteins orient perpendicular to aligned chromosome axes and connect them at a conserved ~100 nm distance, while a distinct, so-called central element substructure exists at the SC’s midline, equidistant from each axis [32]. Zip1, which is predicted to form rod-shaped dimer or tetramer units owing to extensive coiled-coil within the 875-residue protein [33], assembles the transverse filament “rungs” of the yeast SC with its N termini oriented away from chromosome axes and toward one another at the midline of the structure, while the Ecm11-Gmc2 heterocomplex organizes near Zip1’s N termini to generate the central element [34,35].

How do ZMM and SC proteins collaborate to carry out the coordinated processes of meiotic recombination and synapsis? Cytological studies demonstrate that ZMM proteins co-localize at discrete foci on meiotic prophase chromosomes [e.g. 12,16,18,36]; these observations in tandem with genome-wide ChIP assays [37,38] indicate that i) ZMM ensembles localize to sites of DSB repair, ii) ZZS proteins are interdependent on one another for their colocalization to these sites, and iii) Zip1 promotes the localization of Zip3, which then promotes the localization of ZZS proteins followed by MutSγ at these sites. A two-hybrid interaction between Zip4 and Zip3 suggests a mechanism for how Zip3 recruits ZZS to recombination intermediates [37], while the affinity of ZZS and MutSγ heterocomplexes for binding branched DNA structures [37,39–41] and the DNA helicase activity of Mer3 suggest these proteins could play direct roles in generating or stabilizing a DNA joint molecule. The molecular mechanism that underlies SC assembly is unknown, but ZMMs likely function in this process at least in part by recruiting or stabilizing SC structural components at chromosomal sites. For example, the Ecm11-Gmc2 heterocomplex localizes to chromosomes via a direct interaction between Ecm11 and Zip4 [11,42]. Importantly, some interactions involved in recombination and synapsis are likely to be dynamic: The Ecm11-Zip4 interaction is presumably transient during synapsis initiation, in order to allow Ecm11-Gmc2 to assemble the larger SC structure.

Interestingly, Zip1 is a core building block of SC that also has a genetically-separable, crossover-promoting function [28,43–46]. While its key role in promoting crossovers remains unclear, Zip1 is required for Zip3 to be detectable by ChIP at recombination sites [38,46], and crossover-defective but synapsis-proficient *zip1* mutants show multiple phenotypes that together suggest a specific loss of Zip3 activity from DSB sites and from ensembles containing Ecm11 [46]. However, *zip1* mutants exhibit a milder phenotype than *zip3* with respect to Holliday junctions: The *zip1* null or the crossover- and synapsis-defective *zip1[S815A*, *S816A*, *817A*, *S818A]* mutant accumulates a substantial level of double Holliday junction structures after an initial delay, whereas *zip3*, *zip2* and *msh5* meiocytes exhibit little to no evidence of Holliday junction structures [16,28,44]. Thus, while Zip1 may help stabilize Zip3 and other ZMM proteins at DNA repair intermediates, its essential role in crossover formation might be to enforce a particular molecular architecture on these intermediates such that they can interface with resolvase machinery [47].

Our understanding of the physical and functional relationships between individual ZMMs and associated proteins, on and off meiotic chromosomes, remains incomplete. Cytological and ChIP approaches reveal the composition of protein ensembles on chromosomes, while immunoprecipitation followed by mass spectrometry can identify components of relatively high affinity complexes. Here we have used proximity labeling [48] as a complementary approach, to uncover both high and low affinity interactions between components of meiotic ensembles in *S*. *cerevisiae*. Taking advantage of two biotinylated targets detectable on streptavidin blots (Zip3 and Ecm11), we demonstrate that proximity labeling can be used as a phenotyping tool to gain insight into hierarchical relationships between ZMMs, associated meiotic DSB repair factors, and SC proteins. We also use proximity labeling together with mass spectrometry to discover new factors that neighbor known recombination or SC proteins. Taken together, our data underscore the power of proximity labeling for detecting protein ensembles that may not be readily observed through cytological or biochemical methods, and for discovering new potential components of meiotic chromosomal processes.

## Results and discussion

### Several meiotic recombination proteins remain functional when fused to TurboID

To explore proximity interactions between pro-crossover and synapsis proteins in *S*. *cerevisiae* meiotic cells we used a yeast-optimized, engineered version of the *E coli* BirA biotinylase, TurboID [49,50]. For most genes we generated C-terminal fusions between a given meiotic gene and *TurboID-3xMYC*, but an N-terminal fusion was created for *GMC2*, while for *ZIP3* and *ZIP4* the TurboID (but not 3xMYC) was positioned internal to the polypeptide (see Methods).

As for all epitope-tagged fusion proteins, the addition of TurboID may compromise the function of a given bait protein to some extent. However, we observed that most *TurboID* fusion strains show a restoration in meiotic outcome relative to the corresponding null mutant. In our (BR) genetic background, *mer3*, *zip2*, *zip4*, and *spo16* null mutants normally generate few spores [11,51]. We found that while the *MER3-TurboID* strain is defective in spore formation, *ZIP2-TurboID*, *ZIP4iTurboID*, *ZIP4-TurboID*, and *SPO16-TurboID* homozygotes generate an abundance of spores with greater than 88% viability (S1 Fig). Similarly, spores from *MSH5-TurboID*, *MSH4-TurboID*, *ZIP3iTurboID*, and *MLH3-TurboID* homozygotes present better viabilities (82%, 84%, 96%, 92%; S1 Fig) than the corresponding null strains (53%, 71%, 81**%**, 84% respectively; n >700 tetrads). The *ECM11-TurboID* and *TurboID-GMC2* strains show a mild spore viability defect (89%) resembling that of the corresponding null mutants [92%;45].

We furthermore observed that most of the *TurboID* fusion strains assemble SC, as indicated by multiple linear assemblies of Zip1 coincident with Gmc2 on surface-spread meiotic chromosomes from strains homozygous for the *ndt80* null allele, which was used to ensure that a 24 hour sporulation culture is enriched for meiocytes with abundant SC structures [52,53] (S1 Fig). Exceptions are the *MER3-TurboID*, *ECM11-TurboID* and *TurboID-GMC2* homozygotes, which fail to assemble SC. *MER3-TurboID* meiotic nuclei frequently show an aggregate of SC proteins referred to as a polycomplex. The absence of polycomplex in the SC-deficient *TurboID-GMC2* and *ECM11-TurboID* strains is consistent with the fact that both SC and polycomplex structures depend on the Ecm11 and Gmc2 core building blocks [34].

Finally, in most cases anti-MYC antibodies to detect C terminal TurboID fusion proteins labeled dozens of discrete focal structures on mid-meiotic prophase chromosomes, reminiscent of the distribution profile of ZMM proteins (S1 Fig). While Ecm11-TurboID does not support SC formation, the punctate localization of Ecm11-TurboID along meiotic chromosomes may reflect its Zip4-mediated recruitment to recombination sites [42]. Mer3-TurboID, on the other hand, was not detected on chromosomes and only faintly at an SC-based, polycomplex structure. Taken together, these results indicate that most ZMM-TurboID fusion proteins engage functionally with recombination ensembles, at least to some degree. For this exploratory study, we evaluated proximity labeling in all *TurboID* strains created, regardless of whether a particular TurboID fusion appears fully functional.

### Streptavidin blotting reveals biotinylated targets of TurboID-fused meiotic proteins

**Fig 2:**
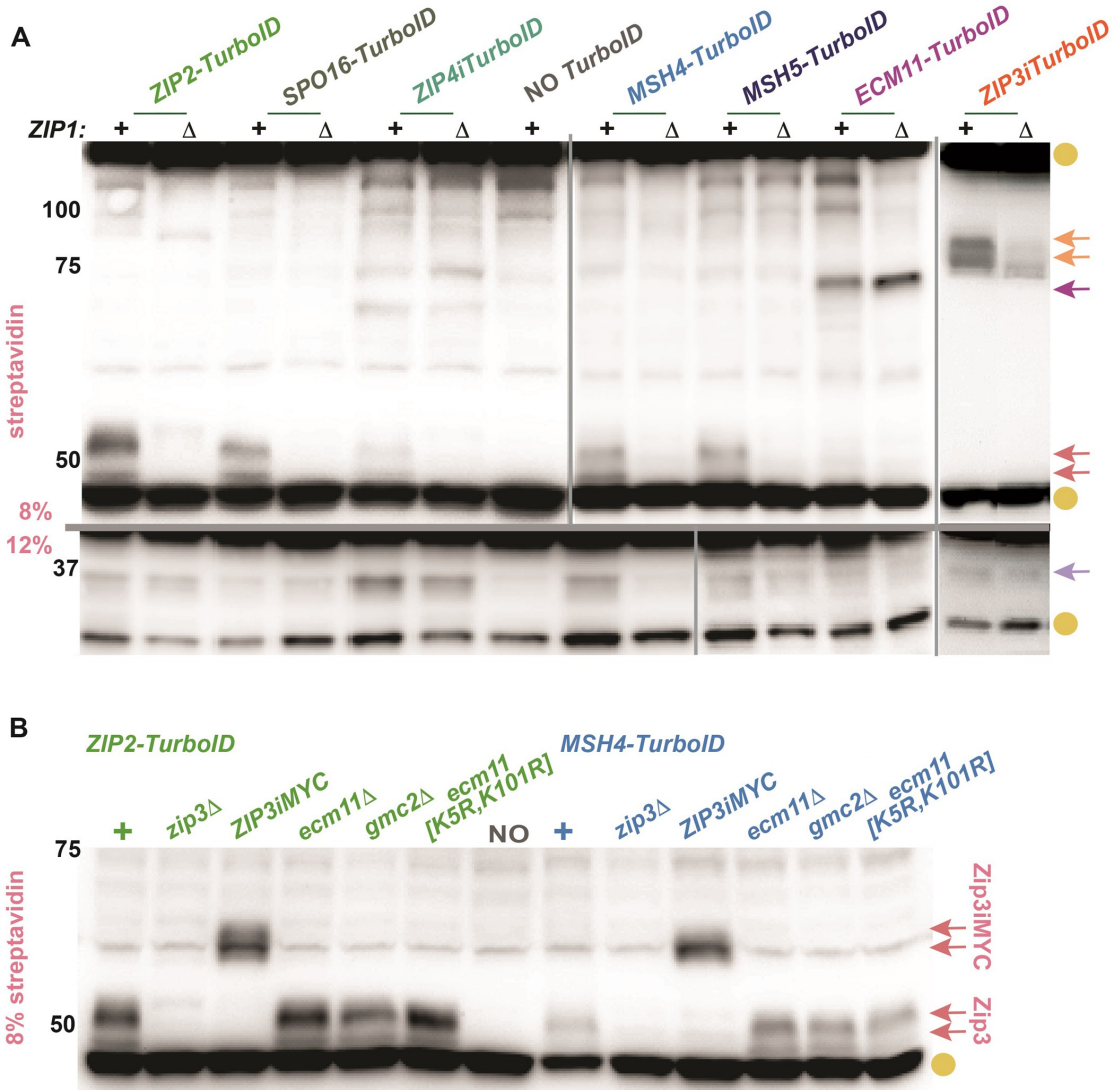
Streptavidin blots can detect proximity labeling targets of ZZS and MutSγ proteins. Proteins extracted from cells arrested at mid-meiotic prophase (strains are homozygous for *ndt80* and collected after 24 hours in sporulation medium) were separated on an 8% or 12% polyacrylamide gel (indicated in pink to left of image), transferred to nitrocellulose and probed with streptavidin:HRP. Blots in (A) show proteins from one of seven distinct *TurboID* fusion strains, which are genotypically either *ZIP1+* or *zip1* (indicated at top of blot). The “NO *TurboID*” lane corresponds to a strain devoid of a *TurboID* transgene. The blot in (B) displays strains homozygous for *ZIP2-TurboID* (green) or *MSH4-TurboID* (blue) that carry various alleles of *ZIP3*, *ECM11* or *GMC2*. Gold circles at the right of blots in (A, B) indicate prominent naturally biotinylated proteins found in meiotic cells independent of TurboID. Arrows in (A, B) indicate the position of biotinylated proteins detected only in strains carrying a particular *TurboID* fusion. Pink arrows in (A, B) correspond to a population of biotinylated Zip3 proteins, which shift to a position of greater mass (B) in *ZIP3iMYC* strains. Dark purple and orange arrows in (A) point to biotinylated targets detected exclusively in *ECM11-TurboID* or *ZIP3iTurboID* strains, respectively; these targets likely correspond to Ecm11-TurboID and Zip3iTurboID protein. Light purple arrow in (A) corresponds to a ~37 kDa protein that is not detected in strains with tagged or null versions of *ECM11* or *GMC2*. Shown are representative data; two biological replicates were examined for all strains. Vertical and horizontal lines demarcate data from independent membranes.

We first asked whether biotinylated targets of TurboID fusions can be observed on a streptavidin blot. Proteins harvested from *TurboID ndt80* cells were separated using 8% or 12% polyacrylamide and streptavidin:HRP was used to detect biotinylated species (Fig 2). As previously observed for yeast mitotic cells [50,54], a small number of naturally biotinylated proteins are detectable even in cells devoid of *TurboID* (Fig 2A, gold circles). However, streptavidin blots also consistently detected biotinylated proteins specific to one or more *TurboID* fusion strains.

**Self-biotinylation of TurboID fusions:** In *ECM11-TurboID* and *ZIP3iTurboID* strains, we observed a specific biotinylated species whose size matched that of the respective TurboID fusion protein (dark purple arrow, orange arrows, Fig 2A). Interestingly, putative Zip3iTurboID self-biotinylation appears reduced when Zip1 is absent, whereas putative Ecm11-TurboID self-biotinylation is not affected by Zip1.

**Zip4-specific targets:** Two targets of Zip4iTurboID with sizes of approximately ~75 and ~70 kDa were repeatedly observed (Figs 2A and S2). Neither Zip4 target depends on Spo11, nor on Zip3, Mer3, Zip1, Ecm11, Gmc2, or the meiotic axis protein Red1 (S2 Fig). The fact that Zip4 proximity labels these targets independent of recombination initiation, ZMMs, and known SC components suggests they are factors associated with the core ZZS (Zip4-Zip2-Spo16) complex. Indeed, the ~75 kDa proximity target may be Zip2 itself, considering that its biotinylation depends on Zip2 and the predicted size of Zip2 is ~83 kDa. Consistent with this possibility, this target appears slightly reduced in abundance when Spo16 is absent, a context in which Zip2 levels diminished [37]. The ~70 kDa target, on the other hand, is biotinylated independent of Zip2 and its identity remains unknown. Using mutant alleles of candidate genes, we determined that the ~70 kDa target is not Rec8, Mek1, Pch2 or Hop1 (S2 Fig).

**Multiple ZMMs proximity label a 45–55 kDa set of proteins:** A group of ~45–55 kDa proximity targets is easily detected in *ZIP2-TurboID*, *SPO16-TurboID*, *MSH4-TurboID*, and *MSH5-TurboID*, but barely detected in *ZIP4iTurboID* and not detected in *ECM11-TurboID* or *ZIP3iTurboID* strains (pink arrows, Fig 2A). Interestingly, proximity labeling of these target proteins depends on Zip1 (Fig 2A).

**A 37 kDa protein:** Finally, we observed a ~37 kDa proximity target in several *TurboID* fusion strains (most easily on blots from 12% gels) and particularly robustly in *ZIP4iTurboID* cells (purple arrow in Fig 2A). This target is likely Ecm11 and will be further discussed below.

### ZMM proteins proximity label Zip3 in a Zip1-dependent but SC-independent manner

We discovered that the 45–55 kDa proximity targets of Zip2-, Spo16-, Msh4- and Msh5-TurboID fusions correspond to the Zip3 protein. Not only do these biotinylated targets depend on Zip3 (Fig 2B), they shift in mass when Zip3 is fused to an internally positioned, 64-residue MYC tag (top-most pink arrows in Fig 2B). Thus, Zip2, Spo16, Msh4, and Msh5 are arranged within the meiotic prophase cell in a configuration that allows the TurboID versions of these proteins to proximity label Zip3 in a Zip1-dependent manner.

A dependence on Zip1 raises the possibility that SC structure is required for the proximity labeling events that target Zip3. This is not the case, as neither Ecm11 nor Gmc2 ‐ key SC structural components -are required for Zip3 proximity labeling by Zip2-TurboID or Msh4-TurboID (Fig 2B).

### Proximity labeling of Zip3 by Zip2-TurboID and Msh4-TurboID is reduced in crossover-defective but not in synapsis-defective *zip1* separation-of-function mutants

Zip1 promotes crossovers via at least one activity that is genetically separable from its role as an SC structural component [45,46]. Because Ecm11 and Gmc2 are dispensable for Zip3 proximity labeling by various ZMM-TurboID fusion proteins, we wondered if Zip1 promotes these Zip3 proximity labeling events through its genetically-separable pro-crossover activity. We explored this possibility by analyzing Zip3 proximity labeling in an array of *zip1* non-null mutant strains.

**Fig 3:**
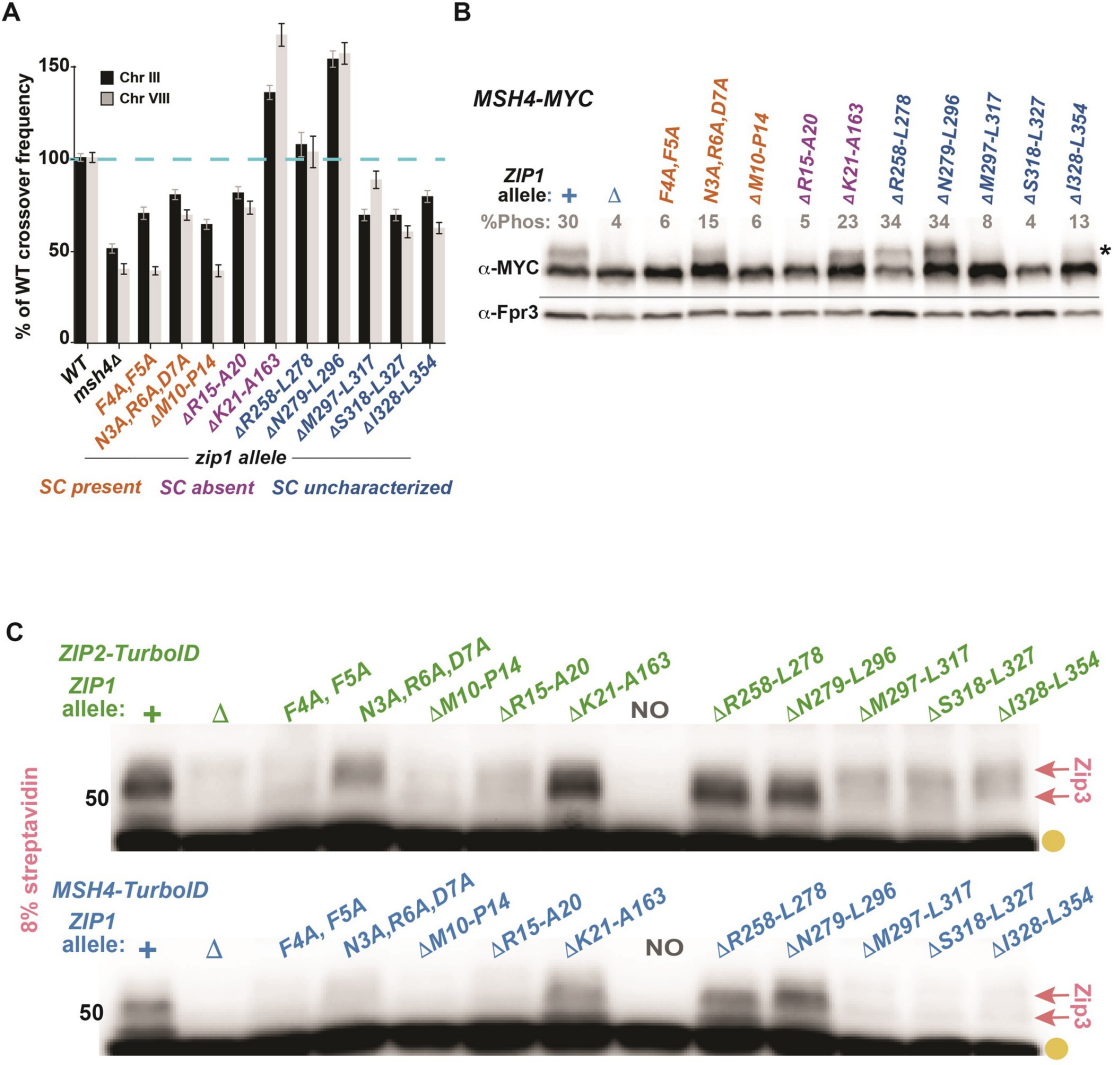
Crossover-defective *zip1* mutants lack detectable proximity labeling of Zip3 by Zip2-TurboID and Msh4-TurboID. Graph in (A) plots crossover recombination frequency on chromosomes III and VIII for several *zip1* mutants; four genetic intervals that span most of the length of III (black bars) and three intervals spanning over half of chromosome VIII (grey bars) are plotted as a percentage of wild type’s cumulative map distance (100%, dotted blue line). Bars indicate standard errors for the cumulative map distances, which were obtained by summing the square product of the standard error for each individual interval, and then calculating the square root of that sum. Most data are calculated from more than 400 tetrads; some of the displayed crossover data has been previously published [45, 46, 56]. See S2 Table for raw data, including standard errors for individual genetic intervals. *zip1* alleles are color-coded to indicate whether they support SC assembly (if known). Western blot in (B) shows Msh4-MYC protein from *ndt80* strains homozygous for *zip1* alleles (cells harvested after 24 hours in sporulation media). Phosphorylated Msh4-MYC protein appears as a slower migrating species, position indicated by an asterisk. Percentage of total Msh4-MYC that corresponds to the phosphorylated version is indicated above each lane (grey); values given are an average of two biological replicates; see S4 Table for raw data. Fpr3 (shown below horizontal line) was used as a loading control. Blots in (C) display biotinylated proteins extracted from *ndt80* cells homozygous for *ZIP2-TurboID* (green) or *MSH4-TurboID* (blue) separated on an 8% polyacrylamide gel. *TurboID* strains are homozygous for *zip1* point mutations or in-frame deletion alleles (listed across top of blots). Gold circles indicate naturally biotinylated proteins, pink arrows correspond to biotinylated Zip3 protein species. Shown are representative blots; two biological replicates were examined for all strains.

We evaluated ten *zip1* alleles, each encoding a protein with a small number of internally deleted or substituted residues within the amino terminal half of the protein (Fig 3). Several of these mutants display a reduction in crossover recombination, as measured genetically using seven intervals that together span nearly the entire length of chromosome III and more than half of chromosome VIII (Fig 3A). Consistent with the prior finding of an association between successful formation of ZMM-mediated recombination intermediates and Msh4 phosphorylation [55], *zip1* alleles showing diminished crossover recombination also display reduced Msh4 phosphorylation (Fig 3B). Other *zip1* alleles promote an excess of MutSγ-dependent crossovers (e.g. *zip1[*Δ*21–163]* or *zip1[*Δ*279–296]*; Fig 3A;[45]. Importantly, four alleles have well-characterized separation-of-function phenotypes: *zip1[F4A*, *F5A]*, *zip1[N3A*, *R5A*, *D6A]*, and *zip1[*Δ*10–14]* mutants show dramatically reduced MutSγ-associated crossovers but robust SC assembly, while *zip1[*Δ*21–163]* exhibits excess MutSγ-dependent crossovers but a failure to assemble SC [46].

Analysis of these *zip1* mutants indicates that proximity labeling of Zip3 by Zip2 and MutSγ correlates with Zip1’s pro-crossover activity and occurs independent of Zip1’s capacity to assemble SC. For example, we observed robust proximity labeling of Zip3 by Zip2-TurboID and Msh4-TurboID in the *zip1[*Δ*21–163]* strain where crossovers are in excess but SC is abolished [45]. The Zip2-Zip3 and Msh4-Zip3 proximity interactions also remain intact in *zip1[*Δ*258–278]* and *zip1[*Δ*279–296]* strains, where crossovers are at a normal or elevated level (Fig 3A and 3C). On the other hand, the Zip2-Zip3 and Msh4-Zip3 proximity interactions are absent in strains homozygous for *zip1[F4A*, *F5A]*, and *zip1[*Δ*10–14]* (Fig 3C), where full SC structures form but MutSγ-dependent crossovers are strongly diminished (Fig 3A and S2 Table, and [45,56]. Consistent with the correlation between ZMM-mediated crossovers and Zip2-Zip3 or MutSγ-Zip3 proximity interactions, meiotic crossovers in *zip1[N3A*, *R6A*, *D7A]*, *zip1[*Δ*15–20]*, *zip1[*Δ*297–317]*, *zip1[*Δ*318–327]*, and *zip1[*Δ*328–354]* homozygotes are intermediate between the low level of *zip1[F4A*, *F5A]* strains and wild-type, and in these strains proximity labeling of Zip3 is clearly reduced (Fig 3C).

Taken together, our data suggest at least one aspect of Zip1’s pro-crossover function is to promote the assembly or maintenance of ensembles that contain Zip3 in proximity to ZZS proteins and MutSγ.

### Zip3’s abundance is uniquely controlled by Zip1

Reduced proximity labeling of Zip3 in *zip1* mutants could reflect a mechanistic defect in assembling Zip3-containing ensembles but could alternatively arise if either the TurboID protein or the target (Zip3) is unstable in the absence of Zip1. We therefore evaluated Zip3’s cellular abundance in *zip1* and several other meiotic mutants 4 and S3A). We found that Zip3iMYC displays a similar abundance to wild type in most *ndt80* strains missing a ZMM or SC protein, but accumulates to only 10–20% of the wild-type level when Zip1 is missing. A time course analysis revealed that Zip3 abundance is sensitive to Zip1’s presence from the earliest time point at which Zip3iMYC is detectable in sporulating cultures (15 hours) and even in the absence of recombination (S3B Fig). We note that the Zip2-Zip3 proximity interaction is first detectable not long after Zip3iMYC is first observed (18 hours; S3C Fig).

Thus, among meiotic recombination and SC proteins, Zip1 is uniquely required for maintaining Zip3’s cellular abundance. To determine if Zip1 influences the abundance of other MutSγ pathway proteins, we examined the level of Msh4 (S4A and S4B Fig). We observed a modest reduction (~50%) in the abundance of Msh4-13xMYC in several mutant strains including *zip1*, *spo11*, *zip2*, *zip3*, *zip4*, *spo16*, *msh5*, *mer3*, consistent with the prior finding that Msh4 is less stable in the absence of *ZMM* function [55]. However, *zip1* mutants did not show a greater diminishment of Msh4 relative to the other mutants. Also, both Zip2-TurboID and Msh4-TurboID appear similarly abundant in *zip1* relative to other meiotic mutants (S5 Fig). We furthermore observed no change in the abundance of epitope tagged Zip4i3xHA in meiotic cells missing Zip1 (S4C Fig).

Finally, we examined Zip1 level in *spo11*, *zip2*, *zip3*, *zip4*, *spo16*, *msh5*, *mer3*, *ecm11*, and *gmc2* strains. We found Zip1 to be no less abundant in *zip3* cells relative to the other mutants (S4A and S6B Fig). Thus, in the meiotic prophase cell, Zip3’s abundance relies on Zip1 but not vice versa.

### Diminished Zip3 in *zip1* mutants does not fully explain the Zip1-dependency of Zip2-Zip3 and MutSγ-Zip3 proximity interactions

**Fig 4:**
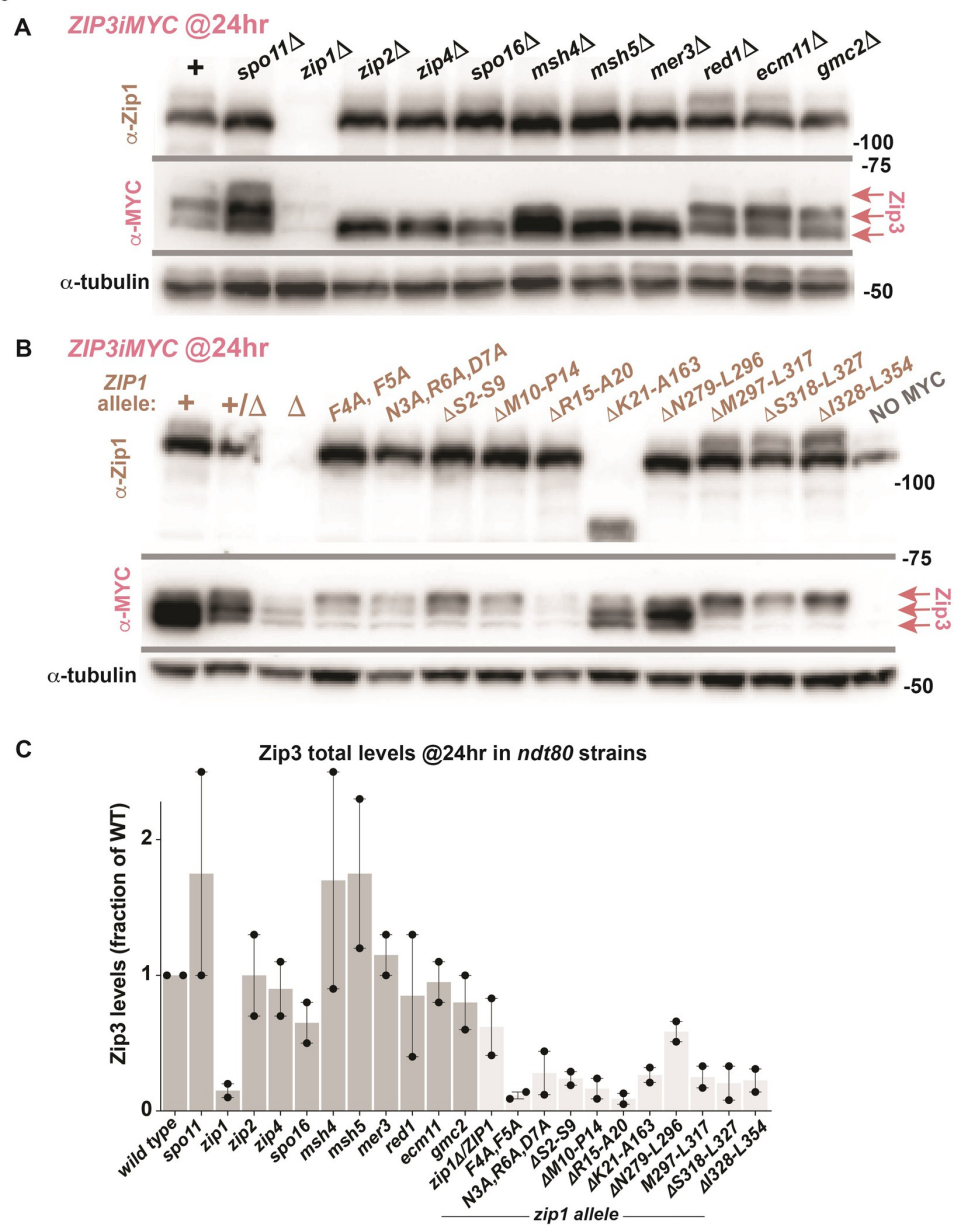
Zip3 abundance relies uniquely on Zip1, and its post-translational modification is promoted by ZMMs. Proteins extracted from mid-meiotic prophase arrested (*ndt80)* cells homozygous for *ZIP3iMYC* were separated on an 8% polyacrylamide gel and transferred to nitrocellulose, which was then sequentially probed with anti-MYC, anti-tubulin, and anti-Zip1 antibodies. Blot in (A) displays protein from various meiotic mutants while blot in (B) shows strains homozygous for specific *zip1* non-null alleles (listed above the blots). Pink arrows in (A, B) indicate Zip3iMYC proteins. Graph in (C) plots total Zip3iMYC protein level in each strain relative to the *ZIP3iMYC* control strain, utilizing tubulin as a loading control (dotted blue bar indicates the level of Zip3iMYC detected in *ZIP3iMYC*, which is set to one). Two biological replicates (black circles) were used to evaluate protein levels; the shaded area represents the mean, and bars indicate range. See S3A Fig for Zip1 levels, S4 Table for raw data.

Considering Zip3’s dependency on Zip1 for its abundance in the meiotic cell, a sub-threshold level of Zip3 (below the minimal level detectable on a western blot) in the *zip1* meiotic cell could perhaps explain the Zip1 dependency of Zip2-Zip3 and MutSγ-Zip3 proximity labeling events. We note, however, that *zip1* non-null mutant strains can show different MutSγ-Zip3 proximity labeling outcomes on a streptavidin blot despite having similarly low Zip3 abundance (Figs 3C and 4B and 4C). For example, Zip3 levels are at ~10%-30% of wild-type in multiple *zip1* mutants where proximity labeling of Zip3 by Zip2-TurboID and Msh4-TurboID is severely diminished or abolished, such as the *zip1[F4A*, *F5A]*, *zip1[*Δ*10–14]*, *zip1[*Δ*297–317]*, *zip1[*Δ*318–327]*, and *zip1[*Δ*328–354]* strains. However, Zip3 levels are also very low (~12%-44%) in the *zip1[N3A*, *R6A*, *D7A]* mutant where the Zip2-Zip3 proximity interaction is readily observed. Most strikingly, Zip3 is at 20%-30% of the wild-type level in the *zip1[*Δ*21–163]* mutant, where both the Zip2-Zip3 and Msh4-Zip3 proximity interactions are robustly observed. These data suggest Zip1 promotes Zip2-Zip3 and MutSγ-Zip3 proximity labeling also through a separate activity, independent of promoting Zip3 abundance *per se*.

We conclude that while Zip1 controls the overall abundance of Zip3 in meiotic cells, Zip1 also may indirectly or directly promote a specific spatial arrangement of Zip3 within recombination ensembles, a spatial configuration that enables Zip2 and MutSγ to proximity label Zip3. In the crossover-proficient *zip1[*Δ*21–163]* mutant, overall Zip3 abundance is low but residual Zip3 is properly positioned to be proximity labeled by Zip2-TurboID and Msh4-TurboID, and to generate MutSγ-facilitated recombination events. For the *zip1[*Δ*10–14]* or *zip1[F4A*, *F5A]* crossover-deficient alleles, Zip3 abundance is similarly low but the absence of Zip2-Zip3 and Msh4-Zip3 proximity labeling in these mutants indicates a second critical defect ‐ the failure of Zip3 to engage properly with recombination ensembles.

### ZMMs promote Zip3 post-translational modification

Our examination of the Zip3 protein in various meiotic mutants unexpectedly revealed that Zip3’s post-translational modification depends upon other ZMMs. Zip3 is modified by phosphorylation [19,38], which explains its appearance as a set of proteins with slightly different masses on a western blot. Interestingly, when *ZIP3iMYC* strains lack Zip2, Zip4, Spo16, MutSγ or Mer3, the size distribution of Zip3 collapses in a manner that indicates a diminishment in post-translational modification (Fig 4A). Strains that lack Spo11, Zip1, Ecm11, Gmc2, or Red1 do not exhibit a similar shift in the size distribution of modified Zip3.

**Fig 5:**
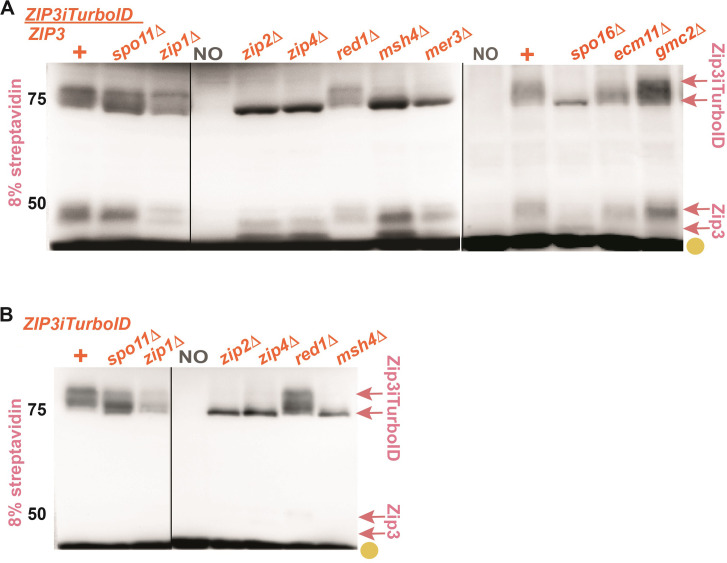
Zip3iTurboID highlights ZMM-mediated post-translational modification of Zip3. Blots display biotinylated proteins extracted from mid-meiotic prophase arrested (*ndt80)* cells and separated on an 8% polyacrylamide gel. Blots contain protein from *ZIP3iTurboID* heterozygotes (A) or *ZIP3iTurboID* homozygotes (B). Strains are also missing the function of one of several genes required for proper MutSγ crossover recombination (indicated above blot). Gold circles indicate naturally biotinylated proteins found in meiotic cells independent of TurboID, pink arrows correspond to biotinylated Zip3 proteins. Vertical lines indicate that lanes were cut from the blot (A, B) or correspond to a distinct blot (A, rightmost section). Shown are representative blots; two biological replicates were examined for all strains. See Fig 9 for a genetic dependency chart which summarizes all genetic dependency data.

Furthermore, we discovered that Zip3iTurboID proximity labels another Zip3 molecule in *trans*, and this analysis led to additional evidence that ZMM proteins indirectly promote Zip3 post-translational modification. *ZIP3iTurboID* heterozygous meiocytes display two sets of biotinylated target proteins, one near the 75 kDa marker (the size of Zip3iTurboID itself) and another corresponding to the sizes of untagged Zip3 (pink arrow, Fig 5A). *trans* proximity labeling of Zip3 occurs independent of Spo11, the ZZS complex, MutSγ, Mer3, Red1, Zip1, Ecm11, and Gmc2. However, the size profiles of biotinylated Zip3 protein (both TurboID-tagged and untagged Zip3) collapse in *zip2*, *zip4*, *msh4*, *mer3*, and *spo16* mutants, in a manner consistent with diminished post-translational modification. Finally, a similar collapse is observed for biotinylated Zip3iTurboID protein in *ZIP3iTurboID* homozygotes missing Zip2, Zip4 or Msh4 protein (Fig 5A and 5B).

Taken together, these findings indicate that phosphorylation of Zip3 relies on ZZS proteins, the Mer3 helicase, and MutSγ.

### Zip2-Zip3 and Spo16-Zip3 proximity interactions require ZZS proteins but not recombination initiation, SC, nor polycomplex structure

Cytological, ChIP, and functional data indicate that ZMM proteins co-localize to the same recombination sites [11,18,37,38,42]. However, ZMMs also localize to sites expected to be devoid of recombination intermediates, such as centromeres and the polycomplex structures that form when SC assembly is delayed or abolished [11,38,46,57–60]. To explore whether proximity labeling of Zip3 is due to interactions within ensembles directly associated with recombination intermediates, we evaluated proximity labeling outcomes of TurboID fusions in strains missing key components of the meiotic recombination pathway.

**Fig 6:**
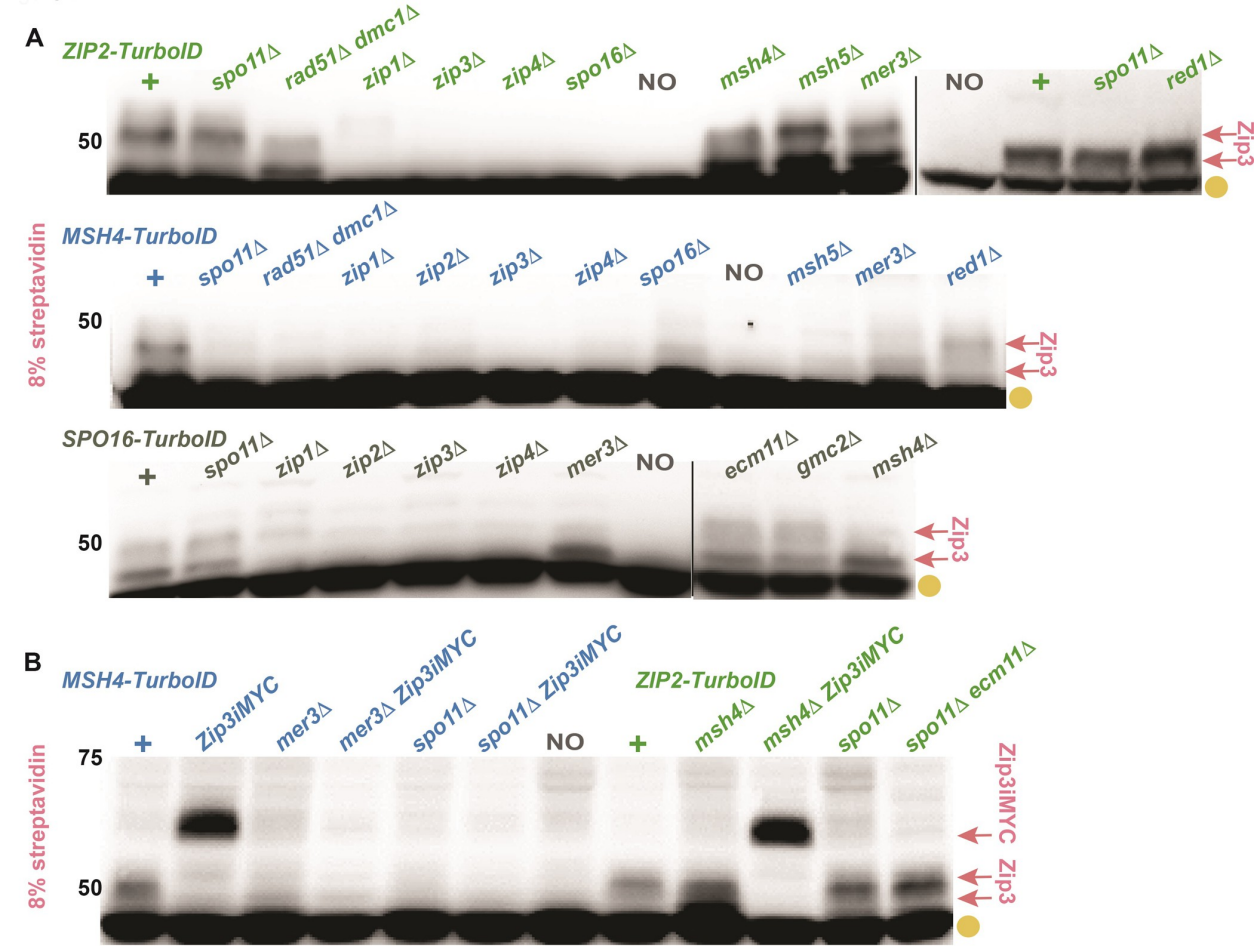
The role of MutSγ meiotic recombination pathway components in Zip3 proximity labeling events. Blots display biotinylated proteins extracted from mid-meiotic prophase arrested (*ndt80)* cells and separated on an 8% polyacrylamide gel. Blots contain protein from *ZIP2-TurboID* homozygotes (A, B; green genotypes), *MSH4-TurboID* homozygotes (A, B, blue genotypes), or *SPO16-TurboID* homozygotes (A, dark green genotypes), missing the function of one of several genes required for proper MutSγ crossover recombination (indicated above blot). Gold circles indicate naturally biotinylated proteins found in meiotic cells independent of TurboID, pink arrows correspond to biotinylated Zip3 proteins. Vertical lines indicate lanes were removed (*SPO16-TurboID*) or correspond to distinct blots (*ZIP2-TurboID*). Shown are representative blots; two biological replicates were examined for all strains. See Fig 9 for a genetic dependency chart which summarizes all genetic dependency data.

We found that ZZS complex proteins are interdependent on one another for their capacity to proximity label Zip3, consistent with prior biochemical data suggesting that Zip2, Zip4, and Spo16 form a stable subcomplex in yeast meiotic cells and the identification of Zip3 among proteins that coimmunoprecipitate with Zip2-TAP [37]. Zip4 and Spo16 are each required for the observed Zip2-Zip3 proximity interaction, while Zip4 and Zip2 are individually required for the Spo16-Zip3 proximity interaction (Fig 6A).

Interestingly, we find that proximity labeling of Zip3 by ZZS proteins occurs even in the absence of recombination. The Zip2-Zip3 proximity interaction occurs independent of Spo11, the Dmc1 and Rad51 recombinases, the Mer3 helicase, MutSγ, and the meiotic axis-associated protein Red1 (Fig 6A). Similarly, Spo11, Mer3, and MutSγ are dispensable for the Spo16-Zip3 proximity interaction. A collapse in the size distribution of biotinylated Zip3 in *ZIP2-TurboID* and *SPO16-TurboID* strains lacking the Rad51 and Dmc1 recombinases, Msh4, Msh5, or Mer3 is consistent with the idea that Zip3 post-translational modification is dependent on ZMM protein function (see above). Zip3iMYC was used to confirm the presence of biotinylated Zip3 in *ZIP2-TurboID msh4* strains because the under-modified Zip3 in this strain migrates very close to the 42 kDa non-specific biotinylated protein (Fig 6B).

In the *spo11* mutant, SC components assemble a polycomplex structure and recombination proteins including ZZS and Zip3 decorate such polycomplexes [e.g. 11]. To determine if the Zip2-Zip3 proximity interaction depends upon polycomplex in the *spo11* mutant, we examined an *ecm11 spo11* double mutant. We observed the Zip2-Zip3 interaction in *ecm11 spo11* cells, demonstrating that it occurs independent of SC or polycomplex structures, even when recombination is absent (Fig 6B).

Taken together, our genetic dependency data suggest that Zip2, Zip3, Zip4, and Spo16 collaborate to form an ensemble configured such that Zip3 can be proximity labeled by Zip2-TurboID or Spo16-TurboID, independent of recombination initiation or SC formation in the meiotic prophase cell. Interestingly, prior studies suggest that ZZS proteins rely on Spo11 for their localization to most chromosomal sites, with the exception of centromere regions [11,16,18,37]. Thus, Spo11- and SC-independent proximity labeling of Zip3 by ZZS may reflect ZZS-Zip3 ensembles off chromosomes or at centromeres.

### The MutSγ-Zip3 proximity interaction requires recombination enzymes that are dispensable for the ZZS-Zip3 proximity interaction

Analysis of *MSH4-TurboID* strains shows that proximity labeling of Zip3 by MutSγ also relies on ZZS proteins (Fig 6A). However, in contrast to the Zip2-Zip3 proximity interaction, the Msh4-Zip3 interaction relies on Spo11, Dmc1 and Rad51 recombinases, and the Mer3 helicase (Fig 6A). Because unphosphorylated Zip3 targets may migrate close to a non-specific 42 kDa biotinylated protein, we used Zip3iMYC to verify the absence of biotinylated Zip3 in *MSH4-TurboID spo11* and *MSH4-TurboID mer3* strains (Fig 6B). Because a diminished abundance of bait protein could impact the amount of target detected, we also confirmed that the level of Msh4-TurboID in *spo11*, *dmc1 rad51*, and *mer3* mutants is similar to the control (S5 Fig). Taken together, our findings suggest the existence of a MutSγ-Zip3 ensemble that depends upon prior formation of a recombination intermediate created through strand invasion, ZZS, and Mer3 helicase activity.

**Fig 7:**
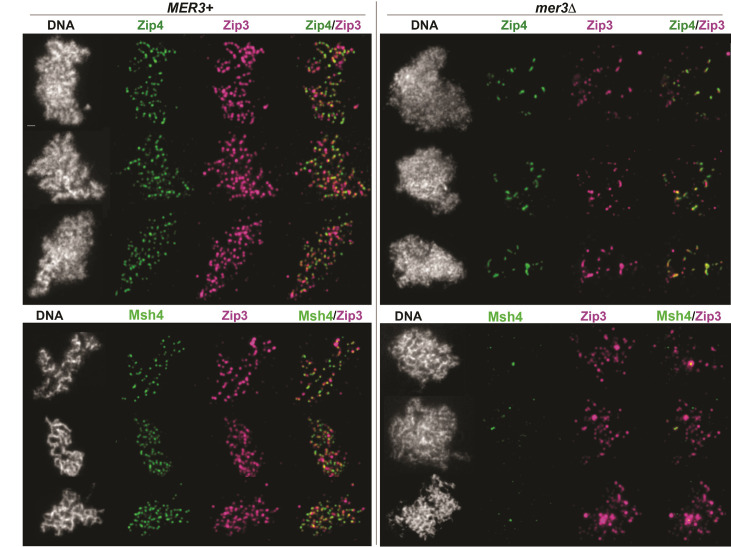
Zip3 colocalizes with ZZS protein Zip4 but not with MutSγ at chromosome-associated structures on mid-meiotic prophase chromosomes when Mer3 helicase is absent. Images show surface-spread chromosomes from strains homozygous for *ndt80* and either *MER3+* (left) or a *mer3* null allele (right). Strains prepared for top panel images carry *ZIP3iMYC* and *ZIP4iHA* alleles, while strains in bottom panel images carry *ZIP3iMYC* and *MSH4-HA* alleles. Zip3iMYC is shown in magenta, while Zip4iHA or Msh4-HA is shown in green; DAPI labels DNA (white). Three distinct nuclei for each genotype are depicted. Bar, 1 micron.

We used immunofluorescence as a second approach to examine the differential reliance of ZZS-Zip3 and MutSγ-Zip3 ensembles on Mer3. ZZS proteins and Msh4 have been found to co-localize with Zip3 at discrete foci on mid-meiotic prophase chromosomes in wild-type cells [46]. Accordingly, in *MER3*+ *ndt80* cells we observe many Zip3iMYC foci co-localized with either Zip4iHA or Msh4-MYC on surface-spread, mid-meiotic prophase chromosomes (Fig 7). *mer3* mutants display fewer Zip3 foci on chromosomes relative to the control, but several large chromosome-associated Zip3 foci are detected and Zip4 co-localizes with Zip3 at these large focal structures (Fig 7, top right). By contrast, hardly any Msh4 protein associates with Zip3 foci or with meiotic chromosomes in *mer3* mutants (Fig 7, bottom right). The detection of a proximity interaction does not necessarily predict cytological detection of co-localized proteins (and *vice versa*), but the cytological observations in this case are consistent with the proximity labeling data, which indicates that Zip2-Zip3 ensembles and Msh4-Zip3 ensembles differentially rely on Mer3. The dependence of the MutSγ-Zip3 proximity interaction on recombination initiation and Mer3 suggests that this interaction takes place within the context of a recombination intermediate.

### Ecm11 is proximity labeled by ZZS proteins and MutSγ

**Fig 8:**
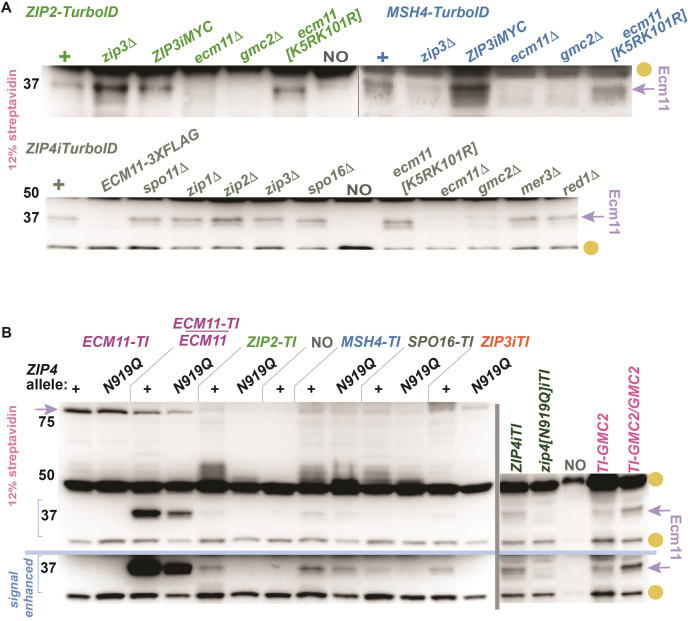
The 37 kDa protein proximity labeled by Zip2-TurboID and Ecm11-TurboID is likely Ecm11. Blots display proteins extracted from *ndt80* cells arrested at mid-meiotic prophase, separated on a 12% polyacrylamide gel and probed with streptavidin-HRP. Gold circles indicate naturally biotinylated proteins, while purple arrows indicate the 37 kDa biotinylated target we believe to be Ecm11, or a shifted species believed to be Ecm11-TurboID (B, left of blot). Blots in (A) show biotinylated proteins from *ZIP2-TurboID* (green genotypes), *MSH4-TurboID* (blue genotypes) or *ZIP4iTurboID* (dark green genotypes) homozygous for alleles of various meiotic recombination genes (indicated at top of blot). Blot in (B) displays biotinylated protein from various *TurboID* fusion strains; some lane pairs in left blot correspond to a particular *TurboID* fusion in the *ZIP4*+ or *zip4N919Q* genetic background (indicated at top of blot), while the right blot shows the *ZIP4iTurboID* and *zip4N919Q-TurboID* strains, as well as a *TurboID-GMC2* homozygote and heterozygote. Independent membranes in (B) are separated by a vertical grey line, and the same blot is shown with computer-enhanced signal below the horizontal blue line. Representative blots are displayed; two biological replicates were examined for all strains. Some of the data informs the genetic dependency chart presented in Fig 9.

Another proximity target of several meiotic TurboID fusions migrates near the 37 kDa marker on a 12% polyacrylamide gel (Fig 2A). The signal for this target is less robust on the streptavidin blot relative to Zip3, but is routinely detected in *ZIP2-TurboID*, *ZIP4iTurboID*, and *MSH4-TurboID* and consistently detected, albeit with a relatively weak signal, in *ZIP3iTurboID*, *SPO16-TurboID* and *MSH5-TurboID* strains. We also observe the 37 kDa target in strains homozygous or heterozygous for *TurboID-GMC2* (Fig 8B). Notably, the 37 kDa biotinylated target is not observed in *ECM11-TurboID* nor in any *TurboID* strains examined that lack Ecm11 or Gmc2 (Figs 2A, 8A and S6), and we show that Zip2-TurboID and Msh4-TurboID proteins remain abundant when Ecm11 is missing (S5 Fig). Analysis of *ecm11[K5R*, *K101R]* homozygotes revealed that Ecm11 SUMOylation is dispensable for proximity labeling of the ~37 kDa target by Zip2-TurboID or Msh4-TurboID (Fig 8A).

The Ecm11 protein interacts directly with Gmc2 and Zip4 [34,42] and has a predicted molecular weight of 34 kD. Thus, the ~37 kDa proximity target is likely to be Ecm11. This possibility is supported by the fact that *ECM11-TurboID* heterozygotes exhibit two biotinylated targets on a streptavidin blot ‐ one corresponding to the size expected of Ecm11-TurboID and another that migrates at the ~37 kDa target position (Figs 8B and S6A and S6B). Furthermore, the 37 kDa target is not detected in a *ZIP4iTurboID* strain homozygous for *ECM11-3xFLAG* [34; Fig 8A] although a shifted biotinylated protein (corresponding to epitope-tagged Ecm11, expected to be 42 kDa) was also not observed. Taken together, our data support either of two distinct possibilities: i) Examined TurboID fusions biotinylate a ~37 kDa protein that is not Ecm11 but depends upon Ecm11 function, or ii) the TurboID fusions proximity label Ecm11, but not Ecm11-3xFLAG. We favor the latter possibility, in part because an amino acid substitution in Zip4 that specifically compromises a yeast two-hybrid interaction with Ecm11 (encoded by *zip4[N919Q]*; [42]) abolishes proximity labeling of the ~37 kDa target by Zip2-, Zip3-, or Spo16-TurboID fusions, and the Zip4[N919Q]iTurboID protein itself fails to proximity label the 37 kDa target (Fig 8B). Hereafter, we refer to the ~37 kDa proximity labeling target as Ecm11.

### Zip1 and Zip3 promote the MutSγ-Ecm11 but not the ZZS-Ecm11 proximity interaction

Consistent with the existence of a direct interaction between Zip4 and Ecm11 [42], Ecm11 is proximity labeled by Zip4iTurboID even when Zip2 or Spo16 is missing (Fig 8A). By contrast, Zip2-TurboID and Spo16-TurboID rely on Zip4 as well as one another to proximity label Ecm11 (S6C and S6D Fig). Zip2 and Spo16 are partially interdependent for their own abundance [37], which may account for their mutual dependence in promoting Ecm11 proximity interactions. A reliance on Zip4 furthermore indicates that the Zip2-Ecm11 and Spo16-Ecm11 proximity interactions depend upon a fully formed ZZS complex, unlike the Zip4-Ecm11 proximity interaction. Finally, our streptavidin blot data indicate that both Zip1 and Zip3 are dispensable for ZZS-Ecm11 proximity interactions in the meiotic cell (Figs 8A and S6C and S6D).

Interestingly, we find that Zip3iTurboID proximity labels Ecm11 in a manner that only partially depends on Zip1 and is independent of Msh4 and Zip4 (S6E and S6F Fig). In light of Zip3’s putative functional capacity as a SUMO E3 ligase and its known role in attenuating Ecm11 SUMOylation [19,34], perhaps the Zip1-independent physical proximity between Zip3 and Ecm11 occurs within an E2/E3(Zip3)/Ecm11 complex.

We also observed that Msh4-TurboID proximity labels Ecm11 in a manner that partially depends on Zip4, and partially depends on Zip1 and Zip3 (S6G Fig). This result raises the possibility that two distinct ensembles facilitate MutSγ proximity to Ecm11: one containing Zip4 and Ecm11, and one that forms independently of Zip4 but contains Zip3. MutSγ has recently been found to be directly targeted by the E2 SUMO conjugase Ubc9 [61]. Thus, perhaps the Zip3-dependent MutSγ-Ecm11 interaction reflects MutSγ joining the E2/E3(Zip3)/SUMO-target(Ecm11) complex mentioned above.

We note that ZZS-Ecm11, Zip3-Ecm11 and Msh4-Ecm11 proximity interactions remain intact in *spo11* mutants (Figs 8A and S6) and thus occur independent of recombination.

### Genetic dependencies of proximity labeling interactions targeting Zip3 or Ecm11: A summary view

**Fig 9:**
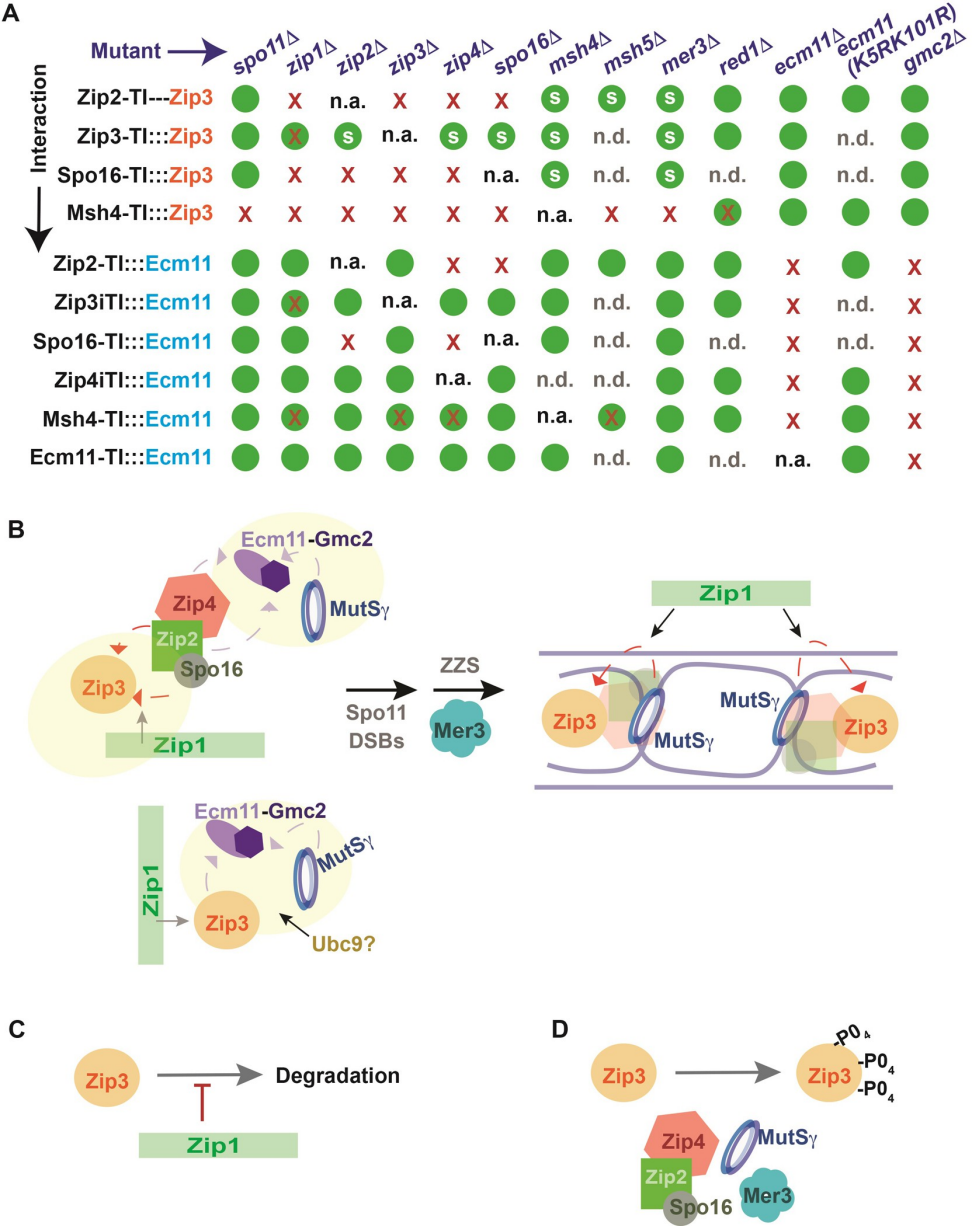
Proximity labeling reports functional relationships between meiotic recombination proteins. (A) Chart illustrates whether a proximity labeling interaction with Zip3 (top four rows) or with Ecm11 (bottom six rows) is robustly detected (green circle), is routinely less robustly detected relative to the control (red X inside a green circle), or not detected (red X) in a particular meiotic mutant (genotypes across top). A green circle with an “s” indicates that the size distribution of biotinylated Zip3 is faster migrating than that of the control. Data plotted is informed by two biological replicates, and multiple technical replicates; representative blots are shown in Figs 2, 3, 5, 6, 8 and S2, and S6). Possible Zip4iTurboID:::Zip3 and Ecm11-TurboID:::Zip3 interactions are not illustrated because the signal to noise corresponding to each of these potential interactions is too low to reliably interpret. n.a. = not applicable; n.d. = not determined. Cartoons in (B-D) illustrate functional relationships inferred from the proximity labeling analysis performed in this study; dotted arrows (orange or purple) indicate *trans* biotinylation of either Zip3 or Ecm11, respectively. (B) illustrates recombination-independent ensembles that support proximity labeling of Zip3 or the Ecm11-Gmc2 heterocomplex (at left): MutSγ independently engages both the Zip4-Ecm11 and Zip3-Ecm11 ensembles that form independent of recombination. As Zip3 is a SUMO E3 ligase that negatively regulates Ecm11 SUMOylation [34] and MutSγ is directly SUMOylated by the Ubc9 E2 [61], we speculate that Ubc9 may be a component of the Zip3-Ecm11-MutSγ ensemble. (B) also illustrates a recombination-dependent ensemble that enables MutSγ to proximity label Zip3 (at right): We propose that MutSγ proximity labels Zip3 at DNA joint molecule recombination intermediates, because in addition to ZZS and Zip1 activity the MutSγ -Zip3 proximity interaction depends on Spo11-mediated DNA double strand breaks, strand invasion, and the Mer3 DNA helicase. (C) illustrates Zip1’s role in maintaining Zip3 abundance in the meiotic cell, while (D) shows the role ZMM proteins play in promoting the post-translational modification of Zip3.

The chart in Fig 9A illustrates genetic dependencies of each proximity interaction involving Zip3 or Ecm11. The data indicate that ensembles containing ZZS proteins and Zip3 exist within meiotic cells independent of recombination and SC structures (and perhaps off chromosomes), while ensembles containing Zip3 in proximity to MutSγ assemble in the physical context of a recombination intermediate. A Zip4-Zip3 two-hybrid interaction and the fact that Zip2-TAP immunoprecipitates Msh5 led De Muyt et al. (2018) to speculate that the ZZS complex might form a bridge between Zip3 and MutSγ at recombination sites [37]. One possibility is that Mer3’s helicase function, in collaboration with other enzymes and ZMMs, influences the three-dimensional structure of the DNA joint molecule in a manner that enables ZZS to bridge a Zip1-Zip3 ensemble with MutSγ (Fig 9B).

Our genetic analysis also suggests the existence of recombination-independent ensembles that contain Ecm11 and either Zip3 or Zip4 (Fig 9B). Zip4 directly interacts with Ecm11 and is required for Ecm11’s localization to meiotic chromosomes [42], but the fact that Zip3 proximity labels Ecm11 independent of Zip4 suggests a parallel molecular mechanism to position ZMM proteins close to SC structural components.

Our findings also suggest that Zip1 not only controls Zip3’s abundance within the meiotic cell (Fig 9C) but also promotes the formation of recombination ensembles where ZZS proteins and MutSγ can properly engage Zip3. This idea stems from the positive correlation found between Zip1’s pro-crossover function and ZZS-Zip3 or MutSγ -Zip3 proximity interactions.

Finally, the genetic dependency data gathered for this study reveals that ZMM proteins promote the post-translational modification of Zip3 (Fig 9A and 9D). The functional relevance of ZMM-dependent Zip3 modification will be the subject of a future study.

### Mass spectrometry identifies shared proximity labeling targets of MutSγ pathway proteins

**Fig 10:**
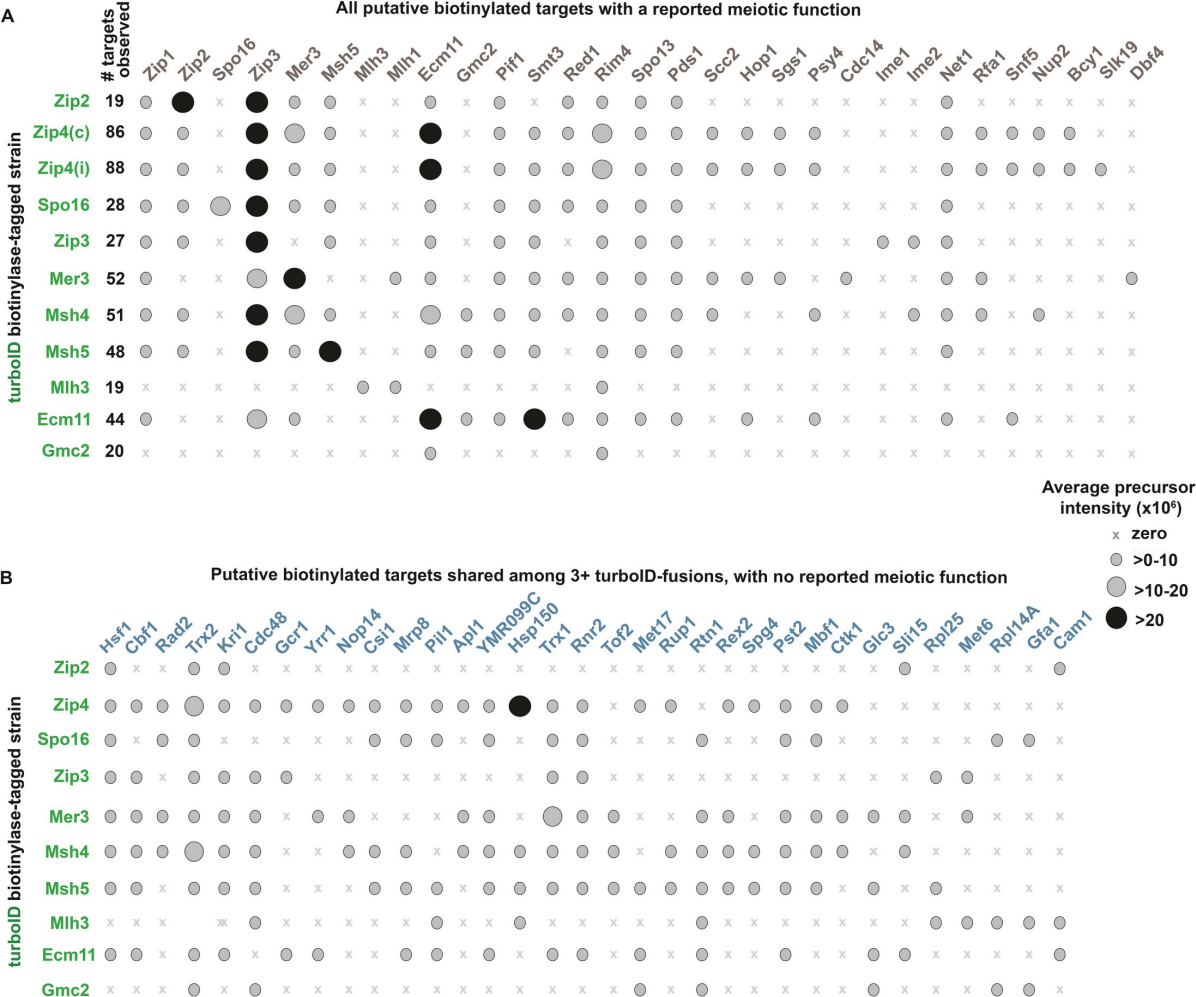
Mass spectrometry reveals additional shared proximity label targets of meiotic recombination associated proteins. Two biological replicates of twelve *ndt80* strains (eleven homozygous for a distinct *TurboID* gene fusion and one devoid of *TurboID*) were sporulated for ~24 hours. Proteins extracted from each strain were incubated with streptavidin-coated beads, beads were washed and processed for ultra-high performance liquid chromatography coupled to tandem mass spectrometry (UPLC-MS/MS) analysis (see Methods). Results were filtered to a 1% false discovery rate at the peptide and protein level using a target-decoy search and a reversed version of the full yeast proteome database. (A, B) Proteins carrying the TurboID fusion are listed on left *y* axis in green, and streptavidin-purified interactors for each TurboID fusion are listed across the top. Note Zip4(c) corresponds to a strain where the TurboID biotinylase is fused to the C terminus of the Zip4 protein, whereas Zip4(i) corresponds to a *ZIP4iTurboID* strain, which encodes a protein containing an internal TurboID. Ovals indicate detection of a particular protein as a streptavidin-purified interactor in a given *TurboID* strain, with the smallest ovals indicating an average precursor intensity of >0–10 (x10^6^), the larger, lightly-shaded ovals indicating an average precursor intensity of between 10 and 20 (x10^6^), and the darkly-shaded large ovals indicating an average precursor intensity of greater than 20 x10^6^; an “x” indicates that the protein was not detected. The total number of targets observed for a given *TurboID* strain is listed in the “# targets observed” column in (A). Note that for most strains, a target was identified in both biological replicates of the *TurboID* strain and not in either biological replicate of the control. For *Zip4iTurboID* or *Zip4-TurboID*, a detected protein was considered a target even if present in only one biological replicate so long as it was detected in both biological replicates of the other strain (and not in either replicate of the control). Similarly, because Msh4-Msh5 and Ecm11-Gmc2 assemble heterocomplexes, a detected protein was considered a target of one component in the heterocomplex even if only present in a single biological replicate so long as it was also detected in both biological replicates of the strain carrying the TurboID fusion of the other component, (and in neither replicate of the control). (A) lists all protein targets identified with a previously reported meiotic function. (B) lists the shared targets of at least three TurboID fusion proteins that have no previously reported meiotic function. In (B), protein targets listed for Zip4 TurboID fusions were identified in both *ZIP4c-TurboID* biological replicates and at least one replicate of *ZIP4iTurboID*, or vice-versa. See S3 Table for the full list of proteins identified by mass spectrometry.

We have described the use of TurboID in yeast meiotic cells as a phenotyping tool to explore genetic dependencies of spatial relationships defined by proximity labeling. To explore whether *TurboID* fusions might identify new factors involved in meiotic processes, we conducted a pull down-mass spectrometry experiment. Total protein was extracted from duplicate cultures of eleven strains, each homozygous for *ndt80* and a given *TurboID* fusion, or a “No TurboID” *ndt80* control strain. Biotinylated proteins were isolated using streptavidin-sepharose beads, subjected to trypsin digest, and analyzed using ultra-high performance liquid chromatography coupled to tandem mass spectrometry (UPLC-MS/MS; see Methods). We removed all proteins identified in either control replicate, and defined *bona fide* proximity labeling targets as those identified in both replicates of a given TurboID fusion (with some exceptions, noted in Fig 10 legend). Using these criteria, we observed 19 targets of Zip2-TurboID, 86 targets of Zip4-TurboID, 88 targets of Zip4iTurboID, 28 targets of Spo16-TurboID, 27 targets of Zip3iTurboID, 52 targets of Mer3-TurboID, 51 targets of Msh4-TurboID, 48 targets of Msh5-TurboID, 19 targets of Mlh3-TurboID, 44 targets of Ecm11-TurboID, and 20 targets of TurboID-Gmc2 (Fig 10 and S3 Table).

As expected, proximity targets of Zip4iTurboID show overlap with targets of Zip4-TurboID (81 shared targets, 7 unique to Zip4iTurboID and 5 unique to Zip4-TurboID; S7 Fig). Furthermore, most proximity targets of Msh4 (43/51) were identified among the 48 targets of Msh4’s heterodimeric partner Msh5 (S7 Fig). These results boost confidence in the success of our pull-down mass spectrometry approach and give a measure of validation to targets defined by a low number of biological replicates (two for each strain). Like Msh4-Msh5, Ecm11 and Gmc2 form a heterocomplex in the meiotic cell [34]. Although neither the Ecm11-TurboID nor TurboID-Gmc2 fusion protein is functional in terms of SC assembly (S1 Fig), 19 of 20 proximity labeling targets identified for TurboID-Gmc2 were also identified as targets of Ecm11-TurboID (S7 Fig).

Consistent with Zip2, Zip4, and Spo16 proteins forming a stable subcomplex [37], 14 targets are shared between Spo16-TurboID and Zip2-TurboID, and 16 of19 Zip2-TurboID targets and 25 of 28 Spo16-TurboID targets are also targets of a Zip4 TurboID fusion protein. Furthermore, Zip2 is a target of Zip2-TurboID, Zip4-TurboID, Zip4iTurboID, and Spo16-TurboID. Spo16 was detected as a target of Spo16-TurboID, but not of Zip2-TurboID nor either of the Zip4-TurboID fusions; the small size of Spo16, however, makes it less likely to be identified in a mass spectrometry experiment. Curiously, Zip4 (a relatively large protein) was not a detected as a biotinylated target of any of the TurboID fusions, even though Zip4 proximity labels several meiotic factors including Zip1, Zip2, Zip3, Mer3, Msh5, and Ecm11. Lysine residues, which serve as a substrate for the biotinylation reaction, occur at a similar frequency in the Zip4 polypeptide chain relative to Zip3 (~8% for both proteins). Perhaps those ZMM-TurboID fusions that exist in ensembles with Zip4 are arranged such that TurboID (typically at the C terminus of the fusion protein) faces “out” toward solvent, while Zip4 engages the non-TurboID end of a given fusion (perhaps the N terminus), making it inaccessible to the biotinylase.

Consistent with cytological evidence for colocalization of meiotic recombination and SC assembly factors at recombination/synapsis sites, many *TurboID* fusion strains share the same proximity labeling targets. For example, Zip3 and Zip1 are targets of every TurboID fusion except for Mlh3-TurboID and TurboID-Gmc2, and Ecm11 was identified as a target of all TurboID fusions except for Mlh3-TurboID (Fig 10A). The SUMO protein (encoded by the *SMT3* gene) was found to be a target of most TurboID fusions, and SUMO peptides identified by mass spectrometry were most abundant in the *ECM11-TurboID* strain, consistent with Ecm11 being one of the most abundant SUMOylated proteins in yeast meiotic prophase cells [62]. These data also show that Msh4-TurboID and Msh5-TurboID (components of MutSγ) proximity label Zip2 as well as the Mer3 helicase, possibly reflecting the engagement of MutSγ with a recombination intermediate.

Another identified target in most *TurboID* fusion strains is the Pif1 helicase. Pif1 localizes to meiotic recombination sites engages with recombination intermediates in a manner that is restrained by Mer3 [63]; our proximity labeling data raise the possibility that Mer3 regulates Pif1 in the physical context of Zip2, Zip4, Spo16, Zip3 and the MutSγ proteins.

Somewhat unexpectedly, Spo13 and Pds1 were identified targets of nearly all TurboID fusions. A dramatic consequence of Pds1 loss during mitosis (a securin protein that binds Esp1/separin and thus protects cohesion from destruction [64]) is premature sister chromatid separation. However, evidence has been reported for Pds1 having a distinct role in meiotic recombination and synaptonemal complex assembly [65]. Spo13 binds the Cdc5 kinase and regulates signaling pathways that govern exit from meiosis after prophase, localizes to centromeres, and regulates kinetochore mono-orientation during meiosis I [66–68]. The fact that Spo13 is a proximity labeling target of several MutSγ pathway pro-crossover proteins in *ndt80* cells raises the possibility that Spo13 plays a role at MutSγ crossover sites, or that ZMM proteins engage with Spo13 at centromeres [38,59,60].

A target of every TurboID fusion is the meiosis-specific mRNA-binding protein Rim4. Rim4 forms amyloid-like aggregates in the cytoplasm of meiotic prophase cells and represses the translation of at least a subset of developmentally regulated mRNAs during meiotic prophase, but may also bind mRNAs that are not translationally repressed [69,70]. An economical explanation for Rim4 being a target of all TurboID fusions examined is that it associates with the mRNA encoding each of these fusions, positioning it in the immediate periphery of TurboID polypeptides as they undergo translation. Another possibility, however, is that Rim4 has a yet undescribed function in ZMM- or SC-associated pathways.

Mlh1, Mlh3, and Rim4 were the only identified meiotic protein targets of Mlh3-TurboID in these experiments, which utilized *ndt80* meiotic cells. The paucity of Mlh3 targets may be explained by the fact that the Mlh1-Mlh3 heterodimer (MutLγ)–mediates resolution of crossover recombination intermediates downstream of Ndt80 activation [51,71].

Importantly, several proteins that do not have a reported role in meiosis were identified as targets of more than one TurboID fusion protein (Fig 10B lists such protein targets shared by at least three different *TurboID* strains). These targets are of particular interest for future study, as at least some of them likely represent factors with meiotic functions that have not yet been investigated.

## Conclusion

Here we report the use of proximity labeling in *S*. *cerevisiae* to explore physical and functional relationships between meiotic proteins involved in the highly coordinated pathways of MutSγ recombination and SC assembly, critical processes for accurate chromosome segregation during meiosis. Many meiotic proteins fused to TurboID are at least partially functional, and we show that these TurboID fusions can biotinylate neighboring proteins without the addition of biotin to growth media, and not only when TurboID is at the N or C terminus, but also when positioned internal to the polypeptide chain.

The covalent modification of a target by TurboID depends on bait-target protein proximity, relative conformations, and the primary amino acid sequence of the target protein itself. Thus, like all tools for reporting a physical relationship between proteins, many protein-protein “proximities” will be missed. The timing of an interaction is also not revealed in these experiments, given that endogenous biotin is utilized. Nevertheless, our data demonstrate that streptavidin blots can be a powerful phenotyping tool to understand the functional requirements for detectable proximity interactions.

We also determined the identities of a broader list of proximity targets biotinylated by our TurboID fusions, through streptavidin pull-down and mass spectrometry. A striking take home from our pull-down data is that the proximity targets of distinct ZMM and SC proteins show substantial overlap, which is consistent with the colocalization of many ZMM and SC proteins on meiotic chromosomes. Furthermore, unexpected biotinylated targets revealed by mass spectrometry present new potentially functional components of meiotic recombination and synapsis pathways to explore.

## Materials and methods

### Strains and crossover data

Strains created for this study are isogenic with the BR1919-8B background [72], and are listed in S1 Table. Knockout alleles and C-terminal *TurboID* fusions were created by standard recombination-based gene targeting procedures. Plasmid pFB1420 (pFA6a-*TurboID-3xMYC-kanMX6*; Addgene) was used to amplify DNA for creating in-frame *TurboID* fusion alleles; note that *3xMYC* follows TurboID in C-terminal fusion alleles, while alleles encoding internal TurboID fusions carry only the four first residues of the 3xMYC tag; the *TurboID* internal fusion alleles and *zip1* and *zip3* non-null alleles were created by *CRISPR*-Cas9-mediated allele replacement as in [46]. Zip3 and Zip4 epitope tags are positioned internal to the gene ORFs (after residue 91 in Zip4 and after residue 245 in Zip3), as described in [11] and notated as *i3xMYC*, *i3xHA* or *iTurboID*. *MSH4-13xMYC* and *MSH4-3xHA* were created using plasmids *pFA6a-13xMYC-kanMX6* and *pFA6a-3HA-kanMX6* respectively [73].

Genetic crossover data was compiled and processed as described in [46].

### Cytological analysis and imaging

Meiotic nuclei from various *ndt80* homozygous strains were surface-spread on glass slides and imaged as described in [45]. The following primary antibodies were used: affinity purified rabbit anti-Zip1 (YenZym Antibodies, LLC, as in [43]; 1:100), mouse anti-cMYC (clone 9E10 Abcam; 1:200), mouse anti-Gmc2 (raised against purified Gmc2, ProSci Inc., 1:800), guinea pig anti-Gmc2_Ecm11 (raised against a co-purified protein complex; ProSci Inc., 1:800), Rabbit anti-HA (Abcam; 1:100), and rabbit anti-Red1 (gift from G.S. Roeder, [74]; 1:200). Secondary antibodies conjugated with Alexa Fluor dyes (Jackson ImmunoResearch) were used at 1:200 dilution. Microscopy and image processing were performed using a Deltavision RT imaging system (General Electric) adapted to an Olympus (IX71) microscope.

### Western and streptavidin blotting

Protein was extracted from 5 mL of sporulating cell culture by TCA precipitation as in [75]; cells were vortexed with glass beads for 10 minutes at 4°C. The final protein pellet was resuspended in 2× Laemmli sample buffer supplemented with 30 mM DTT, at a concentration of ∼20 μg/ μl. Protein samples were heated for 10 minutes at 65°, centrifuged at top speed and ∼100 μg was loaded onto either an 8% or a 12% polyacrylamide/SDS gel. Gels were run either at 80V (for Zip3-iMYC studies) or 100V (for TurboID, Msh4-MYC and Zip4-iHA studies). Protran 0.2μm nitrocellulose (Amersham) was used as the transfer membrane following the manufacturer’s recommendation. Transfer of proteins to nitrocellulose was performed in CAPS pH 11–10% ethanol buffer for TurboID and Zip3-iMYC studies, and Towbin Buffer-10% methanol for MSH4-MYC and Zip4-iHA studies; stir bar and ice pack were used at 100V for transfer. 12% PAGE blots were transferred for 45 minutes whereas 8% PAGE blots were transferred for 1 hour. Membranes were allowed to dry (>30 minutes) after transfer, then washed in 1x PBST buffer. Ponceau S was used to detect total protein and quality of transfer to the membrane, then the membranes were washed twice more with 1x PBST. Membranes for TurboID-biotin studies were blocked using 3% BSA in 1x PBST for 30 minutes and washed once in 1x PBST. Membranes were incubated overnight at 4°C with STAR5B Streptavidin:HRP (BioRad) at a 1:15,000 dilution in 1x PBST. Membranes were then washed three times in 1x PBST and imaged as described below. Membranes for antibody analysis were blocked in 5% non-fat dry milk powder/ 2% BSA in PBST for 30 minutes and washed once in 1x PBST. Membranes were incubated overnight at 4°C with primary antibodies in 1x PBST: Mouse anti-MYC (9E10.3, Abcam) at 1:2000 for Zip3-iMYC, 1:5000 for Msh4-MYC blots; rabbit anti-Zip1 and rat anti-tubulin YOL1/34 (Abcam) at 1:10,000, rabbit anti-Fpr3-C (gift of Dr. Jeremy Thornton) at 1:50,000, mouse anti-HA.11 (Abcam) at 1:1000. Secondary antibodies (HRP-conjugated AffiniPure Donkey anti-rabbit, goat anti-mouse (Jackson ImmunoResearch), and goat anti-rat (Santa Cruz) were used at 1∶10,000 in 1x PBST for 1 hour at room temperature. ECL Prime Western Detection Reagent (Amersham) was used to visualize probes on the membranes; a G:Box mini (Syngene) was used to detect chemiluminescence and ImageJ (https://imagej.nih.gov/ij/index.html) was used to analyze the data.

### Streptavidin pull-down followed by analysis using ultra-high performance liquid chromatography coupled to tandem mass spectrometry (UPLC-MS/MS)

Protein was extracted from ~40 mL of sporulating cell culture at the 24 hour timepoint, using TCA precipitation as described above. TCA preparations are done with 5 mL culture volumes, approximately eight TCA preparations were performed for each strain/replicate (12 strains total with two biological replicates), the excess from an originally 45 mL culture was processed for chromosome spreads to ensure strains entered meiosis successfully. The eight TCA pellets from each strain/replicate were consolidated into one tube using ~975 μL of 2% SDS/Bead Binding Buffer (50 mM Tris-Cl pH 7.5, 150 mM NaCl, 1.5 mM MgCl_2_, 1 mM EGTA, 2% SDS, 1% NP-40, 2 μg/mL sodium deoxycholate, 0.5 mM DTT); protein pellets were heated for two minutes at 65 degrees, disrupted using a P1000 pipette tip, and allowed to rock at room temperature ~30 minutes. Protein solutions were then heated at 65°C for ten minutes, microfuged for 30 seconds at top speed, and the soluble fraction (~950 μL) added to a protein lo-bind microfuge tube (Eppendorf) carrying equilibrated streptavidin-sepharose beads (General Electric # 17-5113-01). A 30 μl volume of streptavidin beads was equilibrated for each sample, via ten washes in 1 mL of RIPA wash buffer (50 mM Tris-Cl pH 7.5, 150 mM NaCl, 1.5 mM MgCl_2_, 1 mM EGTA, 0.1% SDS, 1% NP-40, 2 μg/mL sodium deoxycholate, 0.5 mM DTT). Bead-protein solution was incubated for one hour at room temperature and then overnight at 4°C. Beads were washed three times in 2% SDS wash buffer (50 mM Tris-Cl pH7.5, 2% SDS), then three times on ice in cold RIPA wash buffer, and five times in 1 mL of 20 mM ammonium bicarbonate. Supernatant was removed after the final wash and beads were stored at -80°C until their transport to University of Connecticut’s Proteomics and Metabolomics Facility.

Beads were prepared for UPLC-MS/MS analysis using three washes in 0.1M ammonium bicarbonate, after which cysteine reduction and alkylation was performed using 5 mM dithiothreitol in 0.1M ammonium bicarbonate for 1.5 hour and 10mM iodoacetamide in 0.1M ammonium bicarbonate for 45 minutes in the dark, respectively. Directly afterward, sequencing-grade trypsin (Promega) was added at a 1:20(w/w) enzyme:protein ratio for 16 hour digestion at 37°C. Tryptic peptides were removed with the supernatant, quenched to a final pH of 2.5 using formic acid, then fully desalted using Pierce peptide desalting spin columns (Thermo Scientific) per manufacturers’ instructions. Desalted and dried peptides were resuspended in 0.1% formic acid, quantified, and diluted to 0.3mg/mL. A 1 μL aliquot containing 300ng of peptides was loaded onto a Waters nanoEase m/z Peptide BEH C18 analytical column, separated using a 1-hour UPLC reversed-phase gradient (Solvent A: 0.1% formic acid in water, Solvent B: 0.1% formic acid in acetonitrile), and eluted directly into the Orbitrap Eclipse Tribrid mass spectrometer (Thermo Scientific) using positive mode electrospray ionization. The acquisition method incorporated a TopN data-dependent acquisition mode with a maximum cycle time of 3 seconds. Both MS and MS/MS scans were acquired at high resolution in the Orbitrap mass analyzer. Peptide and protein identifications were achieved by searching the raw data against the full Uniprot reference proteome for *Saccharomyces cerevisiae* (identifier UP000002311, accessed 07/05/2022) plus a custom FASTA database containing the TurboID-tagged protein construct sequences using MaxQuant v1.6.10.43 [76]. Variable modifications included methionine oxidation, acetylation of the protein N-terminus, asparagine or glutamine deamidation, lysine biotinylation, and fixed carbamidomethyl on cysteine residues. All search results were filtered to a 1% false discovery rate at the peptide and protein levels using a target-decoy search. Scaffold 5 (www.proteomesoftware.com) was used to visualize the resulting data, and a protein identification threshold of at least two peptides per protein was used. Protein level quantitation values were calculated as “average precursor intensities”. Listed in S3 Table are the average precursor intensity values for all proteins identified in any of the replicates from a *TurboID* fusion that are not found in either replicate of the *no TurboID* control strain. The mass spectrometry proteomics data have been deposited to the ProteomeXchange Consortium via the PRIDE [77; https://www.ebi.ac.uk/pride/archive/projects/PXD053621] partner repository with the dataset identifier PXD053621.

## Supporting information

S1 Fig Most TurboID fusion proteins support SC assembly, apart from Mer3-TurboID, Ecm11-TurboID, and TurboID-Gmc2. Images show immunofluorescence on surface-spread meiotic chromosomes from *TurboID* strains homozygous for *ndt80*. Each row in both blocks of images corresponds to a different strain, with the top row carrying no *TurboID* and the lower rows homozygous for the *TurboID* fusion indicated at left. The first block of images (four columns) displays surface-spread meiotic nuclei that are each labeled with DAPI (white, first column), anti-Zip1 (green, second column), or anti-Gmc2 (magenta, third column). The fourth column shows the merge of anti-Zip1 and anti-Gmc2. C-terminal TurboID fusion proteins have a 3xMYC epitope, allowing visualization of a particular fusion protein on mid-meiotic prophase chromosomes: Rows in the second block of images display nuclei from the same strain labeled with DAPI, anti-Ecm11-Gmc2 (CE), and anti-MYC (blue, magenta, green, respectively, in the first column), and anti-MYC alone (green) in the second column. Bar, 1 micron. Spore viability for *NDT80+* versions of each *TurboID* fusion strain is listed at right (*n* = number of dissected spores evaluated).
(TIF)

S2 Fig A streptavidin blot detects two proximity labeling targets of Zip4iTurboID. Blots display proteins extracted from meiotic *ZIP4iTurboID ndt80* cells carrying alleles of various meiotic genes (indicated at top of blots). Proteins were separated on 8% polyacrylamide gels and probed with streptavidin-HRP. Gold circles indicate a naturally biotinylated protein, while dark and light green arrows indicate the position of biotinylated proteins specific to Zip4iTurboID. Proximity labeling of the ~75 kDa target (upper dark green arrow) by Zip4iTurboID is absent in the *zip2* mutant and may correspond to Zip2 protein. The ~70 kDa target is observed in all mutants examined; the identity of this target remains unknown. Vertical line demarcates data from independent membranes. Representative blots are displayed; two biological replicates were examined for each strain.
(EPS)

S3 Fig Zip3 abundance relies on Zip1. Graph in (A) relates to Fig 4A and 4B and plots total Zip1 protein in each strain relative to the *ZIP3iMYC* control strain, utilizing tubulin as a loading control (dotted blue bar indicates the level of Zip1 detected in *ZIP3iMYC*, which is set to one). Two biological replicates (black circles) were used to evaluate protein levels; the shaded area represents the mean, and bars indicate range. See S4 Table for raw data. Blot in (B) shows protein extracted from *ndt80 Zip3iMYC* strains at different timepoints during meiotic prophase (10-, 12-, 15-, 18-, 21-, and 24-hour timepoints are listed along the top of each blot), separated on an 8% polyacrylamide gel and transferred to nitrocellulose; the membranes were sequentially probed with anti-MYC, anti-Fpr3, and anti-Zip1 antibodies. Three strains are homozygous for mutant alleles at additional loci: *zip1* (upper right), *spo11* (second row left), *spo11 zip1* (second row right). Pink arrows mark Zip3 proteins. Note that in the second row, lane seven of the blot has a 24-hour wild type control sample for reference. Graphs at the right plot abundance of Zip3 protein at each timepoint, relative to that found in control strains at the 24-hour timepoint (which is set to 1). Fpr3 is used as a loading control for normalization. Two biological replicates (circles) were used to evaluate protein levels; the shaded area represents the mean, and bars indicate range. See S4 Table for raw data. Blot in (C) displays biotinylated Zip3 protein from sporulating cultures of *ZIP2-TurboID ndt80* (green) or *ndt80* (devoid of TurboID) cells, at 10-, 12-, 15-, 18-, 21-, and 24-hour timepoints. Proteins were separated on an 8% polyacrylamide gel, transferred to nitrocellulose and probed with streptavidin:HRP. Gold circle indicates a non-specific, naturally biotinylated protein. Pink arrows indicate biotinylated Zip3 protein. Two biological replicates of the time course gave similar results.
(EPS)

S4 Fig Msh4 and Zip1 levels in mutants missing meiotic recombination or synapsis components. (A) Proteins extracted from *ndt80* cells arrested at mid-meiotic prophase were separated on two 8% polyacrylamide gels and transferred to nitrocellulose; membranes were then probed with either anti-MYC or anti-Zip1 antibodies. Blots show proteins from strains homozygous for *Msh4-MYC* and homozygous for one of several meiotic mutants (listed along the *x* axis above the blot). The slower migrating Msh4 species (indicated by an asterisk) corresponds to phosphorylated Msh4. Percentage of total Msh4-MYC that corresponds to the phosphorylated form is indicated above each lane (grey); values are an average of data from two blots–two biological replicates–see S4 Table for raw data. Fpr3 was detected on each blot (shown below grey line) and utilized as a loading control. Graphs in (B) plot the relative levels of Msh4-MYC and Zip1 protein in each strain, normalized using Fpr3; the level of Msh4-MYC and Zip1 protein in the control strain is set to one and indicated by a dotted blue line. Two biological replicates (circles) were used to evaluate Msh4-MYC levels (left), while two replicates were also used to evaluate Zip1 level (right); shaded areas represent the mean, and bars indicate the range. The membrane in (C) was sequentially probed with anti-HA and anti-Fpr3 antibodies. Graph plots relative levels of Zip4iHA in each strain, normalized using Fpr3; level of Zip4iHA in the control is set to one, indicated by a dotted blue line. Two technical replicates (circles) were used to evaluate Zip4 level; shaded area indicates the mean and bars indicate the range between values. See S4 Table for raw data.
(EPS)

S5 Fig Zip2-TurboID and Msh4-TurboID levels in meiotic mutants. Western blots in (A) show protein extracted from *ndt80* strains carrying either *ZIP2-TurboID* (green), *MSH4-TurboID* (blue) or *ECM11-TurboID* (purple) and homozygous for various meiotic mutant alleles. Extracts were taken at 24-hours of sporulation and separated on an 8% polyacrylamide gel, then transferred to nitrocellulose. Membranes were sequentially probed with anti-MYC then anti-tubulin antibodies. Horizontal lines separate regions of the blot with TurboID fusion protein signal versus tubulin signal. Gold circles indicate a non-specific protein. Graphs in (B) plot abundance of either Zip2-TurboID (left), Msh4-TurboID (center), Ecm11-TurboID (right) in the mutant strains, relative to that found in control strains (set to 1, indicated by a dotted blue line). Tubulin abundance was used as a loading control for normalization. Two biological replicates (circles) were used to evaluate protein levels. The shaded area represents the mean, and bars indicate range; see S4 Table for raw data.
(EPS)

S6 Fig The role of MutSγ meiotic recombination pathway components in Ecm11 proximity labeling events. Blots display biotinylated proteins extracted from mid-meiotic prophase arrested (*ndt80)* cells and separated on a 8% or 12% polyacrylamide gel (indicated in pink). Blots contain protein from strains *ECM11-TurboID* homozygotes (A), *ECM11-TurboID* heterozygotes (B), *ZIP2-TurboID* homozygotes (C), *SPO16-TurboID* homozygotes (D), *ZIP3iTurboID* homozygotes (E), *ZIP3iTurboID* heterozygotes (F), or *MSH4-TurboID* homozygotes (G). Strains on each blot are additionally missing the function of one of several genes required for proper MutSγ crossover recombination (genotypes indicated at top of blot). Gold circles indicate naturally biotinylated proteins found in meiotic cells independent of TurboID, pink arrows correspond to biotinylated Zip3 proteins, and purple arrows correspond to biotinylated Ecm11 proteins. Vertical lines on blots indicate cropping to remove lanes (D), or distinct blots (C, E, F). Shown are representative blots; two biological replicates were examined for all strains. See Fig 9 for a genetic dependency chart which summarizes all genetic dependency data.
(EPS)

S7 Fig Proximity labeling targets of MutSγ pathway proteins substantially overlap. Venn diagrams plot the shared targets, identified by mass spectrometry, of those TurboID fusion proteins that are either distinct fusions involving the same meiotic protein (Zip4iTI versus Zip4cTI) or are known to be part of a stable subcomplex with one another (members of the ZZS complex, the Msh4-Msh5 (MutSγ) heterodimer, or the Ecm11-Gmc2 heterocomplex). “TI” indicates TurboID. Number of targets that are either overlapping or specific to one component are indicated, respectively, in the overlapping or non-overlapping portion of the ovals. Targets specific to a given component (not considered overlapping) are absent in both biological replicates of the TurboID experiments corresponding to the other members of the complex. For comparisons between two proteins, non-overlapping targets are individually listed. See S3 Table for full list of proteins identified by mass spectrometry.
(EPS)

S1 Table Strains used in this study. Strains are of the BR1919-8B background [72].
(PDF)

S2 Table Crossover frequency of select *zip1* alleles. Map distances and genetic interference values were calculated using tetrad analysis or random spore analysis and coefficient of coincidence measurements as described previously [35, 46, 51]. Table gives map distances (standard errors) and their corresponding percentages of the wild-type values for individual intervals, and for the entire chromosome (by summing the intervals on III or VIII). For intervals marked (ND), interference measurements are not obtainable using the coefficient of coincidence method due to an absence of NPD tetrads. Crossover frequencies for strains marked with an *, **, *** were previously published [45, 46, 56, respectively]. Data is plotted on the graph in Fig 3.
(PDF)

S3 Table Streptavidin-purified proteins from 11 *TurboID* fusion strains identified using UPLC-MS/MS. File contains the average precursor intensity data corresponding to *S*. *cerevisiae* proteins detected in streptavidin pull down samples from either replicate of any *TurboID* fusion, and that were also not detected in either of the “no TurboID” replicates. The file contains multiple sheets: The first sheet displays average precursor intensity data for each target on the list across all *TurboID* fusions, while each of the other sheets display average precursor data for proteins detected in at least one replicate of an individual *TurboID* fusion. See Data Availability for link to raw data files.
(XLSX)

S4 Table Raw data for Figs 3B, 4C and S3A, S4 and S5. File contains multiple tabs, linking to spreadsheets that correspond to data in specific figures. Each spreadsheet contains the data used to calculate averages or plotted on graphs in the indicated figures.
(XLSX)
