# Supplementary material for: Proximity labeling reveals new functional relationships between meiotic recombination proteins in S. cerevisiae
Source: PLoS Genet. 2024 Oct 15;20(10):e1011432. doi: 10.1371/journal.pgen.1011432 (PMC11508090; doi:10.1371/journal.pgen.1011432)
Supplement: S1 Table — Strains are of the BR1919-8B background [72]. (PDF) [file pgen.1011432.s008.pdf]

S1 Table.

Strains used in this study. *Strains are of the BR1919-8B background (ROCKMILL AND ROEDER 1998).*

	**GENOTYPE**
YAM1252	*lys2ΔNhe his4-260,519 leu2-3,112 MAT**α** trp1-289 ura3-1 thr1-4 ade2-1*<br>*lys2ΔNhe his4-260,519 leu2-3,112 MAT**a** trp1-289 ura3-1 thr1-4 ade2-1*
JS21	YAM1252 homozygous *ndt80::LEU2*
JS15	YAM1252 homozygous *ZIP2-TurboID-3xMYC-kanMX6 ndt80::LEU2*
JS51	JS15 homozygous *zip1::URA3*
AM4736	JS15 homozygous *zip3::hphMX4*
AM4750	JS15 homozygous *ZIP3-i3xMYC* (3x*MYC* inserted between amino acids 245/246)
AM5601	JS15 homozygous *zip4::kanMX4*
AM4937	JS15 homozygous *ecm11::hphMX4*
AM5024	JS15 homozygous *ecm11-K5R,K101R*
AM5046	JS15 homozygous *gmc2::hphMX4*
AM5332b	JS15 homozygous *rad51::hphMX4 dmc1::kanMX4*
AM4806	JS15 homozygous *spo11::ADE2*
AM5304	JS15 homozygous *spo16::hphMX4*
AM4935	JS15 homozygous *msh4::ADE2*
AM5350	JS15 homozygous *msh4::ADE2 ZIP3-i3xMYC*
AM5000	JS15 homozygous *msh5::kanMX4*
KAV5-1	JS15 homozygous *mer3::hphMX4*
AM6155	JS15 homozygous *spo11::ADE2 ecm11::kanMX4*
AM5093	JS15 homozygous *red1::hphMX4*
AM4747	JS15 homozygous *zip1-F4A,F5A*
AM4746	JS15 homozygous *zip1-N3A,R6A,D7A*
JS53	JS15 homozygous *zip1[ΔM10-P14]*
AM4953	JS15 homozygous *zip1[ΔR15-A20]*
AM5160	JS15 homozygous *zip1[ΔK21-A163]*
AM4961	JS15 homozygous *zip1[ΔR258-L278]*
AM4967	JS15 homozygous *zip1[ΔN279-L296]*
AM5015	JS15 homozygous *zip1[ΔM297-L317]*
AM5033	JS15 homozygous *zip1[ΔS318-L327]*
AM4971	JS15 homozygous *zip1[ΔI328-L354]*
JS27	YAM1252 homozygous *MSH4-TurboID-3xMYC-kanMX6 ndt80::LEU2*
JS83	JS27 homozygous *zip1::URA3*

AM4928	JS27 homozygous *zip2::hphMX4*
AM4742	JS27 homozygous *zip3::hphMX4*
AM4752	JS27 homozygous *ZIP3-i3xMYC*
AM5594a	JS27 homozygous *zip4::kanMX4*
AM4939	JS27 homozygous *ecm11::hphMX4*
AM5040	JS27 homozygous *ecm11-K5R,K101R*
AM4941	JS27 homozygous *gmc2::hphMX4*
AM5334	JS27 homozygous *rad51::hphMX4 dmc1::kanMX4*
AM4805	JS27 homozygous *spo11::ADE2*
AM6075a	JS27 homozygous *spo11::kanMX4 ZIP3-i3xMYC*
AM4930	JS27 homozygous *spo16::hphMX4*
AM4933	JS27 homozygous *msh5::kanMX4*
AM5001	JS27 homozygous *mer3::hphMX4*
AM6072	JS27 homozygous *mer3::hphMX4 ZIP3-i3xMYC*
AM5002	JS27 homozygous *red1::hphMX4*
AM4749	JS27 homozygous *zip1-F4A,F5A*
AM4748	JS27 homozygous *zip1-N3A,R6A,D7A*
JS85	JS27 homozygous *zip1[ΔM10-P14]*
AM5043	JS27 homozygous *zip1[ΔR15-A20]*
AM4735	JS27 homozygous *zip1[ΔK21-A163]*
AM4962	JS27 homozygous *zip1[ΔR258-L278]*
AM4968	JS27 homozygous *zip1[ΔN279-L296]*
AM5039	JS27 homozygous *zip1[ΔM297-L317]*
AM4970	JS27 homozygous *zip1[ΔS318-L327]*
AM4972	JS27 homozygous *zip1[ΔI328-L354]*
K2303	JS27 homozygous *zip4-N919Q*
AM5390	YAM1252 homozygous *SPO16-TurboID-3xMYC-kanMX6 ndt80::LEU2*
K2175	AM5390 homozygous *spo11::ADE2*
K2174	AM5390 homozygous *zip1::URA3*
K2180	AM5390 homozygous *zip2::hphMX4*
K2181	AM5390 homozygous *zip3::URA3*
K2182	AM5390 homozygous *zip4::kanMX4*
K2183	AM5390 homozygous *mer3::hphMX4*
K2313	AM5390 homozygous *ecm11::hphMX4*

K2312	AM5390 homozygous *gmc2::hphMX4*
K2314	AM5390 homozygous *msh4::ADE2*
K2304	AM5390 homozygous *zip4-N919Q*
AM5649	YAM1252 homozygous *ZIP4-iTurboID ndt80::LEU2* (*TurboID* inserted between amino acids 90/91)
K2237	AM5649 homozygous *spo11::ADE2*
K2248	AM5649 homozygous *ECM11-3xFLAG-kanMX4*
K2236	AM5649 homozygous *zip1::URA3*
K2242	AM5649 homozygous *zip2::hphMX4*
K2243	AM5649 homozygous *zip3::URA3*
K2244	AM5649 homozygous *spo16::hphMX4*
K2245	AM5649 homozygous *mer3::hphMX4*
K2246	AM5649 homozygous *ecm11::hphMX4*
K2247	AM5649 homozygous *gmc2::hphMX4*
K2271	AM5649 homozygous *ecm11-K5R,K101R*
K2249	AM5649 homozygous *red1::hphMX4*
K2311	YAM1252 homozygous zip*4-iTurboID-N919Q ndt80::LEU2* (*TurboID* inserted between amino acids 90/91)
JS32	YAM1252 homozygous *MSH5-TurboID-3xMYC-kanMX6 ndt80::LEU2*
JS39	JS32 homozygous *zip1::URA3*
AM5712	YAM1252 homozygous *ECM11-TurboID-3xMYC-kanMX6 ndt80::LEU2*
K2198	AM5712 homozygous *spo11::ADE2*
K2197	AM5712 homozygous *zip1::URA3*
K2199	AM5712 homozygous *zip2::hphMX4*
K2200	AM5712 homozygous *zip3::URA3*
K2201	AM5712 homozygous *zip4::kanMX4*
K2202	AM5712 homozygous *spo16::hphMX4*
K2203	AM5712 homozygous *mer3::hphMX4*
K2204	AM5712 homozygous *gmc2::hphMX4*
K2425	AM5712 homozygous *msh4::ADE2*
K2300	AM5712 homozygous *zip4-N919Q*
AM5713	AM5712 heterozygous *ECM11-TurboID-3xMYC-kanMX6 / ECM11*
K2207	AM5713 homozygous *spo11::ADE2*
K2206	AM5713 homozygous *zip1::URA3*
K2208	AM5713 homozygous *zip2::hphMX4*
K2209	AM5713 homozygous *zip3::URA3*

K2210	AM5713 homozygous *zip4::kanMX4*
K2211	AM5713 homozygous *spo16::hphMX4*
K2212	AM5713 homozygous *mer3::hphMX4*
K2213	AM5713 homozygous *gmc2::hphMX4*
K2205	AM5713 homozygous *ecm11-K5R,K101R*
K2426	AM5713 homozygous *msh4::ADE2*
K2301	AM5713 homozygous *zip4-N919Q*
K2269	YAM1252 homozygous *ZIP3-iTurboID ndt80::LEU2* (*TurboID* inserted between amino acids 245/246)
K2160	K2269 homozygous *zip1::URA3*
K2161	K2269 homozygous *spo11::ADE2*
K2169	K2269 homozygous *zip2::hphMX4*
K2170	K2269 homozygous *zip4::kanMX4*
K2166	K2269 homozygous *mer3::hphMX4*
K2316	K2269 homozygous *ecm11::hphMX4*
K2315	K2269 homozygous *gmc2::hphMX4*
K2423	K2269 homozygous *spo16::hphMX4*
K2167	K2269 homozygous *msh4::ADE2*
K2168	K2269 homozygous *red1::hphMX4*
K2305	K2269 homozygous *zip4-N919Q*
K2270	K2269 heterozygous *ZIP3-iTurboID/ ZIP3*
K2185	K2270 homozygous *zip1::URA3*
K2186	K2270 homozygous *spo11::ADE2*
K2194	K2270 homozygous *zip2::hphMX4*
K2195	K2270 homozygous *zip4::kanMX4*
K2191	K2270 homozygous *mer3::hphMX4*
K2356	K2270 homozygous *ecm11::hphMX4*
K2355	K2270 homozygous *gmc2::hphMX4*
K2424	K2270 homozygous *spo16::hphMX4*
K2192	K2270 homozygous *msh4::ADE2*
K2193	K2270 homozygous *red1::hphMX4*
AM5392a	YAM1252 homozygous *ZIP4-TurboID-3xMYC-kanMX6 ndt80::LEU2*
AM5397a	YAM1252 homozygous *MER3-TurboID-3xMYC-kanMX6 ndt80::LEU2*
AM5396a	YAM1252 homozygous *MLH3-TurboID-3xMYC-kanMX6 ndt80::LEU2*
AM5828	YAM1252 homozygous *TurboID-GMC2 ndt80::LEU2*

AM5829	YAM1252 *TurboID-GMC2/GMC2 ndt80::LEU2*
K842	*lys2ΔNhe HIS4 leu2-3,112 hphMX4@CEN3 MAT**α** ADE2@RAD18 natMX4@HMR*<br>*lys2ΔNhe his4-260,519 leu2-3,112 CEN3 MAT**a** RAD18 HMR*<br>*trp1-289 ura3-1 TRP1MX4@SPO11 spo13::URA3 THR1 210kb ade2-1*<br>*trp1-289 ura3-1 SPO11 SPO13 thr1-4 LYS2@210kb ade2-1*
K852	K842 homozygous *msh4::kanMX4*
K1309	K842 homozygous *zip1-F4A,F5A*
K1281	K842 homozygous *zip1-N3A,R6A,D7A*
SYC107	K842 homozygous *zip1[ΔM10-P14] thr1-4 LEU2@152kbXI 193kb XI*<br>*152kb XI THR1@193kb*
AF8	K842 homozygous *zip1[ΔR15-A20]*
AF6	K842 homozygous *zip1[ΔK21-A163]*
LY674	K842 homozygous *zip1[ΔR258-L278]*
LY582	K842 homozygous *zip1[ΔN279-L296]*
LY579	K842 homozygous *zip1[ΔM297-L317]*
LY583	K842 homozygous *zip1[ΔS318-L327]*
LY584	K842 homozygous *zip1[ΔI328-L354]*
K1268	YAM1252 homozygous *MSH4-13xMYC-kanMX4 ndt80::LEU2*
AM4263	K1268 homozygous *zip1::URA3*
K1840	K1268 homozygous *zip1-F4A,F5A*
K1841	K1268 homozygous *zip1-N3A,R6A,D7A*
AM4269	K1268 homozygous *zip1[ΔM10-P14]*
AM4264	K1268 homozygous *zip1[ΔR15-A20]*
K1838	K1268 homozygous *zip1[ΔK21-A163]*
K2085	K1268 homozygous *zip1[ΔR258-L278]*
K1849	K1268 homozygous *zip1[ΔN279-L296]*
K1850	K1268 homozygous *zip1[ΔM297-L317]*
K1857	K1268 homozygous *zip1[ΔS318-L327]*
K1851	K1268 homozygous *zip1[ΔI328-L354]*
AP184	K1268 homozygous *zip3::hphMX4*
AM5329	K1268 homozygous *spo11::ADE2*
K2003	K1268 homozygous *zip2::hphMX4*
K2006	K1268 homozygous *zip4::hphMX4*
K2000	K1268 homozygous *spo16::hphMX4*
AM4510	K1268 homozygous *msh5::kanMX4*
AM4506	K1268 homozygous *mer3::hphMX4*

AM4524	K1268 homozygous *ecm11::kanMX4*
K1988	K1268 homozygous *gmc2::hphMX4*
K1770	YAM1252 homozygous *ZIP3-i3xMYC ndt80::LEU2*
K1884	K1770 homozygous *spo11::ADE2*
K1771	K1770 homozygous *zip1::URA3*
K1976	K1770 homozygous *zip2::hphMX4*
K1979	K1770 homozygous *zip4::hphMX4*
K1973	K1770 homozygous *spo16::hphMX4*
AM5351	K1770 homozygous *msh4::ADE2*
K1958	K1770 homozygous *msh5::hphMX4*
K1961	K1770 homozygous *mer3::hphMX4*
K1970	K1770 homozygous *red1::hphMX4*
K1952	K1770 homozygous *ecm11::hphMX4*
K1955	K1770 homozygous *gmc2::hphMX4*
K2461	K1770 homozygous *zip4-N919Q*
K1772	K1770 homozygous *zip1::URA3*/ *ZIP1*
K1777	K1770 homozygous *zip1-F4A,F5A*
K1778	K1770 homozygous *zip1-N3A,R6A,D7A*
K1774	K1770 homozygous *zip1[ΔS2-S9]*
K1775	K1770 homozygous *zip1[ΔM10-P14]*
K1776	K1770 homozygous *zip1[ΔR15-A20]*
K1773	K1770 homozygous *zip1[ΔK21-A163]*
K1791	K1770 homozygous *zip1[ΔN279-L296]*
K1792	K1770 homozygous *zip1[ΔM297-L317]*
K1793	K1770 homozygous *zip1[ΔS318-L327]*
K1794	K1770 homozygous *zip1[ΔI328-L354]*
AM6122	K1770 homozygous *spo11::ADE2 zip1::URA3*
K1795	YAM1252 homozygous *ZIP4-i3xHA ndt80::LEU2* (*3xHA* inserted between amino acids 90/91)
K1796	K1795 *zip1::URA3*
AM6257	YAM1252 *ZIP4-i3xHA*/*ZIP4* homozygous *ZIP3-i3xMYC ndt80::LEU2*
AM6259	AM6257 homozygous *mer3::hphMX4*
AM6071	YAM1252 *ZIP3-i3xMYC*/*ZIP3* homozygous *MSH4-3xHA-kanMX4 ndt80::LEU2*
AM6059	AM6071 homozygous *mer3::hphMX4*
