# Supplementary material for: Proximity labeling reveals new functional relationships between meiotic recombination proteins in S. cerevisiae
Source: PLoS Genet. 2024 Oct 15;20(10):e1011432. doi: 10.1371/journal.pgen.1011432 (PMC11508090; doi:10.1371/journal.pgen.1011432)
Supplement: S2 Table — Map distances and genetic interference values were calculated using tetrad analysis or random spore analysis and coefficient of coincidence measurements as described previously [35, 46, 51]. Table gives map distances (standard errors) and their corresponding percentages of the wild-type values for individual intervals, and for the entire chromosome (by summing the intervals on III or VIII). For intervals marked (ND), interference measurements are not obtainable using the coefficient of coincidence method due to an absence of NPD tetrads. Crossover frequencies for strains marked with an *, **, *** were previously published [45, 46, 56, respectively]. Data is plotted on the graph in Fig 3. (PDF) [file pgen.1011432.s009.pdf]

S2_Table. Crossover frequency of select *zip1* alleles

GENOTYPE (STRAIN)	INTERVAL (CHROMOSOME)	PD	TT	NPD	TOTAL	cM (± SE)	%WT	cM by chrm	%WT by chrm	NPDobs/NPDexp (± SE)	viability
*WT****	*HIS4-CEN3* (III)	584	528	10	1122	**26.2 (1.1)**	**100**	103.7 (III)	**100**	0.20 (0.07)	
(K842)	*CEN3-MAT* (III)	708	416	4	1128	**19.5 (0.9)**	**100**			0.15 (0.08)	**97%**
	MAT-RAD18 (III)	412	676	18	1106	**35.4 (1.3)**	**100**			0.16 (0.04)	
	RAD18-HMR (III)	651	454	8	1113	**22.6 (1.0)**	**100**			0.24 (0.09)	
	SPO11-SPO13 (VIII)	453	630	33	1116	**37.1 (1.6)**	**100**	75.2 (VIII)	**100**	0.40 (0.07)	
	SPO13-THR1 (VIII)	913	180	2	1095	**8.8 (0.7)**	**100**			0.48 (0.34)	
	THR1-LYS2 (VIII)	490	590	8	1088	**29.3 (1.0)**	**100**			0.11 (0.04)	
*msh4Δ**	*HIS4-CEN3* (III)	375	96	1	472	**10.8 (1.1)**	**41**	53.4 (III)	**51**	0.35 (0.35)	
(K852)	*CEN3-MAT* (III)	425	51	1	477	**6.0 (0.9)**	**31**			1.36 (1.36)	**71%**
	MAT-RAD18 (III)	276	184	7	467	**24.2 (1.9)**	**68**			0.55 (0.21)	
	RAD18-HMR (III)	352	116	0	468	**12.4 (1.0)**	**55**			ND	
	SPO11-SPO13 (VIII)	365	89	3	457	**11.7 (1.4)**	**32**	30.4 (VIII)	**40**	1.20 (0.70)	
	SPO13-THR1 (VIII)	423	27	0	450	**3.0 (0.6)**	**34**			ND	
	THR1-LYS2 (VIII)	319	129	2	450	**15.7 (1.4)**	**54**			0.34 (0.24)	
*zip1-F4A,F5A***	*HIS4-CEN3* (III)	297	114	3	414	**15.9 (1.6)**	**61**	72.4 (III)	**70**	0.61 (0.36)	
(K1309)	*CEN3-MAT* (III)	300	114	2	416	**15.1 (1.5)**	**77**			0.41 (0.29)	**87%**
	MAT-RAD18 (III)	260	148	6	414	**22.2 (2.0)**	**63**			0.67 (0.28)	
	RAD18-HMR (III)	272	142	3	417	**19.2 (1.7)**	**85**	29.2 (VIII)	**39**	0.38 (0.38)	
	SPO13-THR1 (VIII)	373	34	0	407	**4.2 (0.7)**	**48**			ND	
	THR1-LYS2 (VIII)	304	101	2	407	**13.9 (1.5)**	**47**			0.52 (0.38)	
*zip1-N3A,R6A,D7A***	*HIS4-CEN3* (III)	304	219	3	526	**22.5 (1.4)**	**86**	83.1 (III)	**80**	0.18 (0.11)	
(K1281)	*CEN3-MAT* (III)	367	156	1	524	**15.5 (1.1)**	**79**			0.14 (0.14)	**93%**
	MAT-RAD18 (III)	264	255	5	524	**27.2 (1.6)**	**77**			0.20 (0.09)	
	RAD18-HMR (III)	354	171	3	528	**17.9 (1.4)**	**79**			0.33 (0.19)	
	SPO11-SPO13 (VIII)	289	223	5	517	**24.5 (1.6)**	**66**	51.8 (VIII)	**69**	0.28 (0.13)	
	SPO13-THR1 (VIII)	462	52	0	514	**5.1 (0.7)**	**58**			ND	
	THR1-LYS2 (VIII)	292	224	1	517	**22.2 (1.2)**	**76**			0.06 (0.06)	
*zip1[ΔM10-P14]***	*HIS4-CEN3* (III)	411	139	5	555	**15.2 (1.5)**	**54**			0.94 (0.43)	**92%**
(SYC107)	*CEN3-MAT* (III)	443	119	5	567	**13.1 (1.4)**	**75**	68.5 (III)	**64**	1.37 (0.62)	
	MAT-RAD18 (III)	336	219	5	560	**22.2 (1.5)**	**57**			0.33 (0.15)	
	RAD18-HMR (III)	365	196	1	562	**18.0 (1.1)**	**79**			n.d.	
	SPO11-SPO13 (VIII)	399	149	2	550	**14.6 (1.2)**	**48**	(VIII)	**39**	0.32 (0.23)	
	iTHR1-iLEU2 (XI)	515	47	0	562	**4.2 (0.6)**	**46**			n.d.	
*zip1[ΔR15-A20]***	*HIS4-CEN3* (III)	409	139	4	552	**14.8 (1.4)**	**56**	84.2 (III)	**81**	0.75 (0.38)	
(AF8)	*CEN3-MAT* (III)	388	172	7	567	**18.9 (1.6)**	**97**			0.84 (0.32)	**87%**
	MAT-RAD18 (III)	304	232	14	550	**28.7 (2.1)**	**81**			0.78 (0.22)	
	RAD18-HMR (III)	348	199	7	554	**21.8 (1.7)**	**96**			0.58 (0.22)	
	SPO11-SPO13 (VIII)	380	169	6	555	**18.5 (1.6)**	**50**	55.0 (VIII)	**73**	0.73 (0.30)	
	SPO13-THR1 (VIII)	434	89	0	523	**8.5 (0.8)**	**97**			NA	
	THR1-LYS2 (VIII)	275	239	9	523	**28.0 (1.9)**	**96**			0.43 (0.15)	
*zip1[Δ21-163]***	*HIS4-CEN3* (III)	263	311	10	584	**31.8 (1.8)**	**121**	139.9 (III)	**135**	0.28 (0.09)	
(AF6)	*CEN3-MAT* (III)	242	331	13	586	**34.9 (1.9)**	**179**			0.30 (009)	**89%**
	MAT-RAD18 (III)	208	329	17	554	**38.9 (2.2)**	**110**			0.35 (0.09)	
	RAD18-HMR (III)	231	320	11	562	**34.3 (1.9)**	**152**			0.26 (0.08)	
	SPO11-SPO13 (VIII)	182	332	44	558	**53.4 (3.2)**	**144**	124.5 (VIII)	**166**	0.88 (0.16)	
	SPO13-THR1 (VIII)	325	194	3	522	**20.3 (1.4)**	**231**			0.24 (0.14)	
	THR1-LYS2 (VIII)	162	329	34	525	**50.8 (3.0)**	**173**			0.59 (0.12)	

S2_Table. Ctnd

GENOTYPE (STRAIN)	INTERVAL (CHROMOSOME)	PD	TT	NPD	TOTAL	cM (± SE)	%WT	cM by chrm	%WT by chrm	NPDobs/NPDexp (± SE)	viability
zip1[ΔR258-L278]	*HIS4-CEN3* (III)	42	65	1	108	**32.9 (3.4)**	**126**	111.4 (III)	**107**	0.10 (0.10)	
(LY674)	*CEN3-MAT* (III)	76	*35*	0	111	**15.9 (2.2)**	**82**			ND	**97%**
	MAT-RAD18 (III)	34	71	2	107	**38.8 (4.1)**	**110**			0.12 (0.10)	
	RAD18-HMR (III)	61	45	1	107	**23.8 (3.5)**	**105**			0.29 (0.29)	
	SPO11-SPO13 (VIII)	47	59	3	109	**35.3 (4.9)**	**95**	77.1 (VIII)	**103**	0.42 (0.26)	
	SPO13-THR1 (VIII)	87	19	1	107	**11.7 (3.3)**	**133**			2.08 (2.11)	
	THR1-LYS2 (VIII)	43	65	0	108	**30.1 (2.4)**	**103**			ND	
zip1[ΔN279-L296]	*HIS4-CEN3* (III)	192	300	17	509	**39.5 (2.4)**	**151**	158.6 (III)	**153**	0.39 (0.10)	
(LY582)	*CEN3-MAT* (III)	141	374	11	526	**41.8 (1.9)**	**214**			0.34 (0.04)	**89%**
	MAT-RAD18 (III)	171	313	25	509	**45.5 (2.8)**	**129**			0.48 (0.11)	
	RAD18-HMR (III)	230	270	9	509	**31.8 (1.9)**	**141**			0.29 (0.10)	
	SPO11-SPO13 (VIII)	130	329	40	499	**57.0 (3.4)**	**154**	117.3 (VIII)	**156**	0.55 (0.13)	
	SPO13-THR1 (VIII)	327	152	2	481	**17.1 (1.4)**	**194**			0.26 (0.18)	
	THR1-LYS2 (VIII)	135	332	14	481	**43.2 (2.3)**	**147**			0.29 (0.03)	
zip1[ΔM297-L317]	*HIS4-CEN3* (III)	399	118	2	519	**12.5 (1.2)**	**48**	71.5 (III)	**69**	0.50 (0.36)	
(LY579)	*CEN3-MAT* (III)	388	134	3	525	**14.5 (1.3)**	**74**			0.57 (0.33)	**84%**
	MAT-RAD18 (III)	308	184	7	499	**22.7 (1.8)**	**64**			0.60 (0.23)	
	RAD18-HMR (III)	332	167	9	508	**21.8 (2.0)**	**96**			1.00 (0.34)	
	SPO13-THR1 (VIII)	373	106	1	480	**11.7 (1.1)**	**133**			0.29 (0.29)	
	THR1-LYS2 (VIII)	263	206	12	481	**28.9 (2.3)**	**99**			0.73 (0.22)	
zip1[ΔS318-L327]	*HIS4-CEN3* (III)	377	100	2	479	**11.7 (1.3)**	**45**	71.5 (VIII)	**69**	0.65 (0.47)	
(LY583)	*CEN3-MAT* (III)	363	118	3	484	**14.1 (1.4)**	**72**			0.69 (0.40)	**81%**
	MAT-RAD18 (III)	271	194	7	472	**25.0 (1.9)**	**71**			0.49 (0.19)	
	RAD18-HMR (III)	306	159	6	471	**20.7 (1.8)**	**92**			0.67 (0.28)	
	SPO11-SPO13 (VIII)	354	113	3	470	**13.9 (1.4)**	**37**	45.3 (VIII)	**60**	0.73 (0.43)	
	SPO13-THR1 (VIII)	399	54	0	453	**6.0 (0.8)**	**68**			ND	
	THR1-LYS2 (VIII)	249	201	5	455	**25.4 (1.8)**	**87**			0.30 (0.14)	
zip1[ΔI328-L354]	*HIS4-CEN3* (III)	363	137	4	504	**16.0 (1.5)**	**61**	81.7 (III)	**79**	0.69 (0.35)	
(LY584)	*CEN3-MAT* (III)	344	168	1	513	**17.0 (1.2)**	**87**			0.11 (0.11)	**90%**
	MAT-RAD18 (III)	266	231	12	509	**29.8 (2.2)**	**84**			0.60 (0.18)	
	RAD18-HMR (III)	341	162	5	508	**18.9 (1.6)**	**84**			0.60 (0.27)	
	SPO11-SPO13 (VIII)	343	146	4	493	**17.2 (1.5)**	**46**	46.3 (VIII)	**62**	0.58 (0.30)	
	SPO13-THR1 (VIII)	417	70	0	487	**7.2 (0.8)**	**82**			ND	
	THR1-LYS2 (VIII)	289	195	3	487	**21.9 (1.5)**	**75**			0.22 (0.13)	

Data previously published:

* VOELKEL-MEIMAN et al. 2016

** VOELKEL-MEIMAN et al. 2019

*** VOELKEL-MEIMAN et al. 2022
